# *Clostridioides difficile* meets the adenosine system: the art of manipulating host homeostasis

**DOI:** 10.1186/s12929-025-01160-8

**Published:** 2025-07-11

**Authors:** Katia Fettucciari, Luigi Cari, Andrea Spaterna, Rachele Del Sordo, Filippo Tavanti, Pierfrancesco Marconi, Gabrio Bassotti

**Affiliations:** 1 Biosciences & Medical Embryology Section, Department of Medicine and Surgery, University of Perugia, 06129 Perugia, Italy; 2 Pharmacology Section, Department of Medicine and Surgery, University of Perugia, 06129 Perugia, Italy; 3 School of Biosciences and Veterinary Medicine, University of Camerino, 62024 Matelica, Italy; 4 Anatomic Pathology and Histology Section, Department of Medicine and Surgery, University of Perugia, 06129 Perugia, Italy; 5 Gastroenterology, Hepatology & Digestive Endoscopy Section, Department of Medicine and Surgery, University of Perugia, 06129 Perugia, Italy; 6 Gastroenterology & Hepatology Unit, Santa Maria Della Misericordia Hospital, 06129 Perugia, Italy

**Keywords:** *Clostridioides difficile* infection, *Clostridioides difficile*toxin A, *Clostridioides difficile* toxin B, Adenosine, Colon pathophysiology, Inflammation, Anti-inflammatory response, Immune cells, Enteric glial cells

## Abstract

**Background:**

Adenosine is a ubiquitous endogenous molecule capable of influencing several pathophysiological aspects. The adenosine system is extremely complex, starting from the generation of intracellular and extracellular adenosine, the regulation of its levels, and its action on four different receptors that vary in affinity and distribution in the different cell types and tissues.

The most relevant effects of adenosine during infections and inflammation are documented on all types of immune cells, including those of adaptive immunity (T lymphocytes, B lymphocytes, regulatory cells) and of natural immunity (macrophages, polymorphonuclear cells, dendritic cells, natural killer).

Of interest, the adenosine system is also strongly involved in the pathophysiology of colonic cells.

*Clostridioides difficile (C. difficile),* responsible for 15–20% of all cases of antibiotic-associated diarrhea, is an infection that has been evolving over the past two decades due to the unstoppable spread of *C. difficile* in the anthropized environment and the progressive human colonization. The pathological activity of *C. difficile* is due to toxin A (TcdA) and B (TcdB) which profoundly alter the homeostasis of the adenosine system, acting both at the level of its generation and on the expression and regulation of adenosine receptors. The final effect consists in an attenuation of the inflammatory response to favor the persistence of the *C. difficile* infection.

**Conclusion:**

This review highlights a new ability of *C. difficile,* through its Tcds, of manipulating the host to its advantage.

## Background

Adenosine (ADO), a ubiquitous endogen molecule, influences some fundamental pathophysiological aspects. The mechanisms of ADO production, regulation of levels, and effects mediated through ADO receptors are so complex and articulated that it is considered as a system (ADO system). In physiological conditions, there are very low concentrations of extracellular ADO (40–80 nM). In situations of severe alteration of homeostasis such as tissue stress, (e.g. necrosis or apoptosis), hypoxia, and inflammation, ADO concentrations increase and can reach the micromolar range.

In the gastrointestinal tract, particularly in the colon, ADO performs regulatory functions on all the basic activities of this apparatus.

The colon is also the site of infections by several bacteria. One of the most important gastrointestinal infections is that caused by *Clostridioides difficile* (formerly *Clostridium difficile*, *C. difficile*), an opportunistic pathogen that is progressively colonizing humans, representing approximately 30% of all gastrointestinal infections. *C. difficile* is the most common cause of antibiotic-associated diarrhea.

The pathological manifestations of *C. difficile* are mainly due to the production by *C. difficile* of two exotoxins (Tcds), toxin A (TcdA) and toxin B (TcdB), that induce both in vitro and in vivo inactivation of the Rho-GTPase by glucosylation causing cytopathic and cytotoxic effects which lead to the loss of many important biological functions. However, Tcds can cause also cytotoxic effects that are glucosylation-independent, and production/secretion of chemokines and proinflammatory cytokines.

*C. difficile*, through its two main Tcds, TcdA, and TcdB, interacts with some molecules of the ADO system and influences the course of infection, an event involving the cells that are subject to the activity of the Tcds without undergoing cell death. This issue requires a re-assessment of the possible effects of this interaction, effects that can have both immediate and predictable consequences in the time course and which are the aims of this review.

## General characteristics of the adenosine system

ADO is an endogenous ubiquitous molecule that influences almost all aspects of cellular physiology [1–3], and it is composed of an adenine molecule linked to a ribose (deoxyribose) via a β-N9-glycosidic bond (ATP-derived nucleoside) [4]. Under physiological conditions, extracellular levels of ADO range between 20 and 300 nm. In situations of severe alteration of homeostasis, the concentration can even reach the micromolar range [1].

**Fig. 1:**
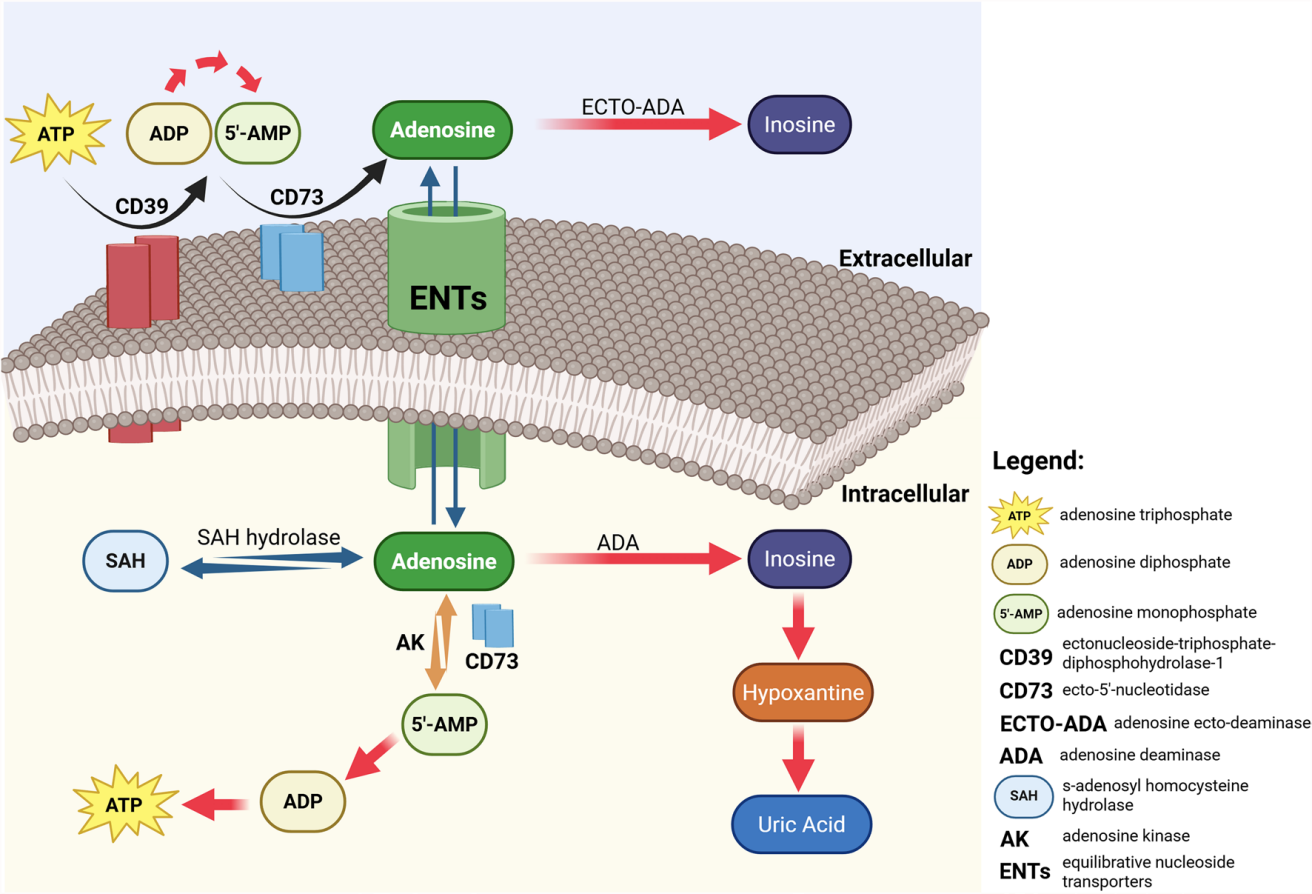
Adenosine metabolism and transport in intracellular and extracellular milieu. ADO can be produced from extracellular ATP by the sequential action of the CD39 and CD73 ecto-enzymes expressed on the outside of the cell membrane. Intracellular ADO synthesis can occur by the action of SAH hydrolase acting on SAH and by the action of AK acting on 5'AMP in conjunction with intracellular CD73 ecto-enzyme. Of course, the intracellular and extracellular ADO transport systems, the ENTs, act predominantly on the concentration gradient of ADO. Finally, ADO levels are regulated intracellularly and extracellularly by the action of ADA, which converts ADO into inosine. "Created in BioRender https://BioRender.com/wnmewx7"

The main mechanism responsible for the extracellular generation of ADO is the dephosphorylation of its precursors: adenosine triphosphate (ATP), adenosine diphosphate (ADP), and adenosine monophosphate (AMP) (Fig. 1) [1–6]. These precursors are released by different cell types under stress conditions [1–5]. The generation of ADO occurs through the sequential action of the ectoenzymes ectonucleoside-triphosphate-diphosphohydrolase-1 (NTPDase1, CD39) and endo-5'-nucleotidase (CD73) (Fig. 1) [7, 8]. In physiological conditions, intracellular ADO originates via hydrolysis of AMP by endocellular CD73 and hydrolysis of S-adenosyl homocysteine (SAH) by SAH hydrolase (Fig. 1) [1, 2, 6]. Once generated, ADO becomes extracellular through the action of SCL28 family of cation-linked concentrative nucleotide transporters (CNT) and SLC29 family of energy-independent equilibrative nucleoside transporters (ENT) [9] that allow ADO to pass freely across the membrane (Fig. 1). The direction of its release from cells or its uptake is determined by differences in the concentration of ADO across the membrane. The role of ENT in this transfer is more critical than that of CNT. In fact, the 4 isoforms of ENT (ENT 1–4) transport nucleotides in or out of the membrane based on the concentration of ADO, while the 3 isoforms of CNT (CNT 1–3) favor the influx of ADO against a concentration gradient using the Na^+^ ion gradient as a source of energy (Fig. 1) [1–6, 9].

Normally, the flow of ADO is from the extracellular to the intracellular environment, whereas during hypoxia the opposite occurs. After intracellular uptake, ADO undergoes deamination to inosine by adenosine deaminase (ADA), or phosphorylation to AMP by adenosine kinase (AK), and has a physiological half-life of < 1 s. AK plays a physiological major role in clearance, whereas in pathological conditions where the increase in ADO is rapid, control of its levels occurs by deamination through ecto-adenosine deaminase (ECTO-ADA) or by ENT-mediated influx (Fig. 1) [1–5].

**Fig. 2:**
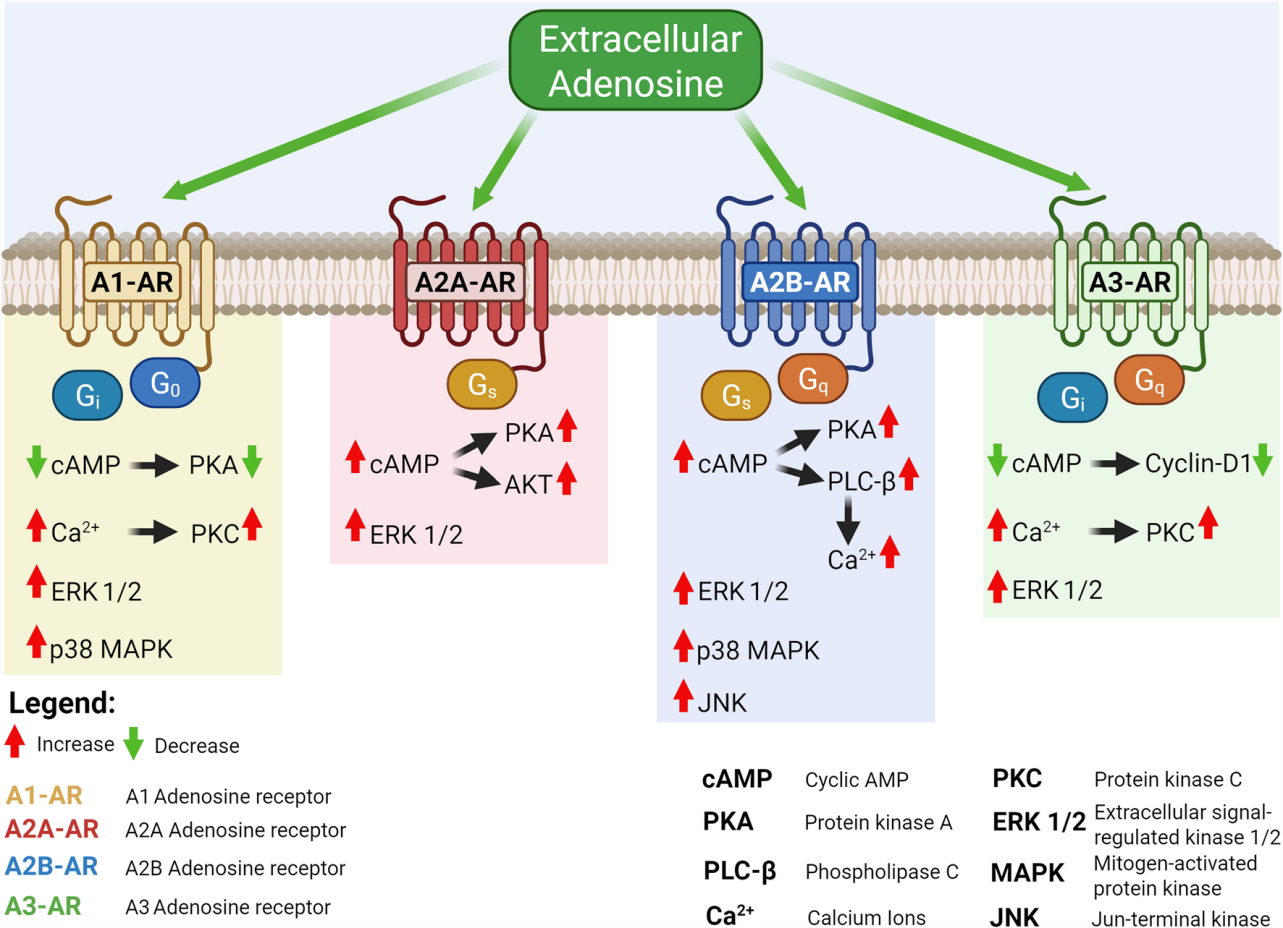
ARs and second messengers signalling pathway activated by extracellular adenosine. Each of the four ARs is characterized by the activation of characteristic signal pathways that define the quality of the cellular response. Some common molecules stimulated by ADO are, activation of MAPKs such as p38, ERK1/2, stimulation of increased intracellular Ca^2+^ levels, cAMP activation, and activation of PKC and activation or inhibition of PKA. Of course, the final outcomes of AR activation depend on the interaction of the signaling pathways and the type of cell involved and its state. "Created in BioRender https://BioRender.com/b01cpt5"

ADO mediates its physiological effects through the activation of four adenosine receptors (ARs) characterised by a different tissue distribution and effector coupling (G-proteins) and affinity for ADO: high-affinity A1-AR, A2A-AR, A3-AR and low-affinity, A2B-AR (Fig. 2) [3, 10–12].

ECTO-ADA is capable of modulating the binding of ADO to the four ARs of ADO, binding to them, and increasing their receptor affinity and signalling.

### Intracellular formation of Adenosine

Beyond ADO generation by the hydrolysis of AMP by endocellular CD73 and of SAH by SAH-hydrolase, other events that contribute to the generation of ADO are the hydrolysis of AMP by cytoplasmatic-5-nucleotidase-I (cN-I) and the degradation of ATP to AMP by nucleotide pyrophosphatase/phosphodiesterase (NPP), followed by the action of CD73 that then gives origin to ADO.

Further, nicotinamide adenine dinucleotide (NAD +) released from the savage pathway is hydrolysed by CD38 to ADP-ribose, which is then further degraded to AMP by ectonucleotide pyrophosphatase/phosphodiesterase family member 1 (CD203α). At this point, CD73 can act to dephosphorylate AMP to ADO [1–9].

### Extracellular formation of Adenosine

The main mechanism for the extracellular generation of ADO is the dephosphorylation of its precursors, ATP, ADP, and AMP. These precursors are released by different cell types under stress conditions and under the action of the ectoenzymes CD39 and CD73 form ADO (Fig. 1) [1, 4–6].

In conclusion, the main ways of generating ADO are (Fig. 1) [1–6]:

1. Consecutive dephosphorylation of extracellular (e) ATP catalysed by CD39 and CD73 with the sequence:
2. CD39 dephosphorylate eATP to ADP;
3. CD73 dephosphorylate ADP to AMP and finally to ADO.
4. NAD + released by the savage pathway is hydrolysed by cyclic ADP ribose hydrolase (CD38) to ADP-ribose, which is then further degraded to AMP by CD203α. At this point, CD73 intervenes and dephosphorylates AMP to ADO.
5. Production of intracellular ADO by hydrolysis of AMP by cN-I.
6. Production of intracellular ADO by hydrolysis of AMP by SAH hydrolase.
7. Production of ADO from eATP by the action of NPP which degrades it to AMP which then by the action of CD73 forms ADO.

### Adenosine level regulation

The two key events that regulate intracellular and extracellular levels are mediated by ADA and AK.

Indeed ADA, after intracellular uptake of ADO, induces deamination to inosine. Furthermore, extracellular ADA acts on extracellular ADO. Instead, AK phosphorylates ADO to AMP [1–5, 13]. Due to these two mechanisms, the physiologic half-life of ADO is less than 1 s.

ADA activity is an oxygen-dependent process and is therefore inhibited in a hypoxic environment.

ADA is ubiquitous in the cells of all tissues. It belongs to the family of hydrolases that catalyse cleavage by reaction with H_2_O and catalyses the reaction in which 5-adenosine monophosphate (5-AMP) plus H_2_O produces 5-inosine monophosphate (5-IMP) and NH_3_. 5-IMP is then catalysed by inosine-5-monophosphate dehydrogenase (IMPDH) into guanine nucleotide.

AK plays a major role in decreasing ADO in a physiological environment, through phosphorylation of ADO to AMP. In non-physiological conditions, where there is a rapid increase in ADO, control of its extracellular levels occurs through deamination by ECTO-ADA, or through influx mediated by ENT, which can transfer ADO both into and out of the cell, whereas CNT transports ADO only within the cell, i.e., only in one direction. Transport of ADO outside the cell can also take place via exosomes that not only contain ADO but also CD39 and CD73 [1–9].

## Adenosine receptors and their functions

ADO mediates its effects through the activation of four ARs, divided into three classes: the first class is constituted by A1-AR, the second class by A2A-AR, A2B-AR, and the third class by A3-AR [3, 10–12]. ARs have different tissue and cellular distribution and are characterised by different effector coupling molecules that account for the different physiological effects. The receptors also vary in their degree of affinity for ADO, which is high for A1-AR, A2A-AR, and A3-AR, and low for A2B-AR (Table 1) [3, 10–12]. ARs have a molecular organisation characterised by a common structure consisting of a core domain that crosses the cell membrane seven times, in which each helix of 20–27 amino acids is linked by three intracellular and three extracellular loops [3, 10–12]. The extracellular NH_2_-terminal part contains one or more glycosylation sites. The intracellular COOH-terminal part contains phosphorylation and palmitoylation sites and plays an important role in receptor desensitisation and in the internalisation mechanism [3, 10–12]. There are several G-protein-coupled receptors (GPCRs) that are bound to ARs, which in turn can be present as homomers, oligomers, and heteromers. GPCRs heteromers are signalling entities, characterised by different functional properties compared to that of ARs homomers (Table 1) [3, 10–12]. For instance, the A1-AR-A2-AR adenosine receptor unit is a molecular complex that consists of two different GTP-dependent protein (G-protein)-coupled receptors, with A1-AR being coupled to G protein of the inhibitory type (Gi1) and A2-AR being coupled to G-stimulatory protein (Gs) (Table 1) [3, 10–12]. Coupling two different G-proteins allows the heterodimer to activate opposite (contrasting) signals that act on the intracellular cyclic adenosine monophosphate (c-AMP)-dependent pathway. This complex unit represents a membrane sensor of ADO concentration, being able to discriminate between low and high levels of ADO [3, 10–12]. Indeed, when ADO levels are low, its interaction occurs preferentially with the A1-AR protomer of the heteromer and activates the Gi/o protein (Table 1), thus reducing the levels and/or activity of adenylyl cyclase/adenylate cyclase (AC), protein kinase (PK)A, and the uptake of γ -amino-butyric-acid (GABA). On the other hand, when ADO levels are higher, binding to the A2-AR component of the complex is favoured, thus reducing activation of A1-AR, via the Gs protein that is associated with the AC/cAMP/PKA cascade, which ultimately has the effect of increasing GABA uptake. Finally, the phenomenon of heteromerisation is also present with A3-AR receptor forming homodimers and heterodimers with A1-AR, i.e. forming the A1-AR-A3-AR complex [3, 11, 12].

**Table 1:** ARs and Immune Cells: Mechanism of Action and Distribution

	A1-AR	A2A-AR	A2B-AR	A3-AR
Adenosine affinity	1–10 nM	30 nM	1000 nM	100 nM
G protein coupling	Gi/o	Gs	GsGq	GiGq
Effector system				
AC	↓	↑	↑	↓
cAMP	↓	↑	↑	↓
PLC	↑	–	↑	↑
K^+^—Ca^2+^	↑	–	↑	↑
PKC	↑	–	↑	↑
PKA	﻿↓	↑	↑	↓
MAPKs	↑	↑	↑	↑
PI3K	↑	-	-	↑
Distribution in main immune cells				
PMN	X	X	X	X
Eosinophil	X	–	–	–
Monocytes	X	X	–	X
Macrophages	X	X	X	X
DC immature	X	–	–	X
DC mature	–	X	X	X
Mast Cells	–	X	X	X
Natural Killer	–	X	X	X
T Cells	–	X	X	X
Treg Cells	–	X	–	–
B Cells	X	X	–	X
Breg Cells	X	X	–	–

↑ Increase; ↓ Decrease; X: Indicated the Expression

### A1-AR

The A1-AR receptor is coupled with Gi1 which acts on AC (Table 1) [3, 11, 12]. Consequently, its activation by ADO causes a decrease in the intracellular concentration of cAMP (second messenger). A1-AR also induces activation of phospholipase C (PLC)-β, leading to an increase in inositol 1,4,5-trisphosphate (IP3) and intracellular Ca^2+^ levels, which stimulate the Ca^2+^-dependent PKC and/or other Ca^2+^ binding proteins (Table 1 and Fig. 2). It is also involved in the intracellular phosphorylation cascade of the mitogen-activated protein kinase (MAPK) family that includes ERK1/2, p38, and JNK1/2 (Table 1 and Fig. 2) [3, 11, 12]. A1-AR is expressed in neutrophils, eosinophils, monocytes-macrophages (Table 1) where it essentially promotes pro-inflammatory effects [3, 11, 12].

### A2A-AR

The A2A-AR receptor couples to a Gs that increases cAMP production, the effects of which are then mediated by cAMP-dependent kinase (PKA) and some MAPKs such as p38, ERK1/2 and JNK1/2 (Table 1 and Fig. 2) [3, 11, 12]. A2A-AR is expressed in the cells of the immune system (Table 1), particularly in leukocytes, platelets, and vascular epithelium, where it mediates numerous anti-inflammatory, anti-aggregating, and vasodilator effects. Its activation has as its main effect the activation of PKA-cAMP-dependent (Table 1 and Fig. 2), which in turn activates by phosphorylation numerous proteins including phosphodiesterase, cAMP-responsive element-binding protein (CREB), DARPP-32 (dopamine- and cAMP-regulated neuronal phosphoprotein) [3, 11, 12]. This receptor is also involved in the modulation of MAPK signalling. A2A-AR can also interact with different accessory proteins: D2-dopamine receptors, alpha-actin, ADP-rybosylation factor nucleotide site opener (ARNO), ubiquitin-specific-protease-4 (USP4), translin-associated protein X (TRAX), which mutually influence each other in their molecular functions [3, 11, 12].

### A2B-AR

The A2B-AR receptor production couples to a Gs and increases cAMP, the effects of which are then mediated by PKA and some MAPKs (Table 1 and Fig. 2). Furthermore, unlike A2A-AR, it can couple to another G protein, called Gq (Table 1 and Fig. 2) [3, 10–12]. The Gq protein stimulates the catabolism of phosphoinositides (membrane phospholipids) which then, via two second messengers, leads to the mobilisation of intracellular Ca^2+^ stores and to the activation of certain lipid-dependent kinases (PKCs) that are themselves MAPK-sensitive, i.e. activated by MAPKs: p38, ERK1/2, and JNK1/2 (Table 1 and Fig. 2). This receptor is highly expressed in immune cells (Table 1 and Fig. 2), endothelial cells, neurons, and microglia and in tissues. Its expression is upregulated under conditions such as hypoxia, inflammation, and cellular stress. The signalling pathways of A2B-AR involve the activation of AC via Gs proteins leading to the phosphorylation of PKA and the involvement of different cAMP-dependent effectors such as exchange proteins that are directly activated by cAMP (Table 1 and Fig. 2). In addition, it can regulate ion channels through their βγ subunits. A2B-AR has multiple binding patterns that modulate its responses and functions: Netrin-1, E3KARPP, Ezirin, PKA, SNARE, NF-κB1/P105 and α-actinin-1 [3, 10–12]. Some examples of these interactions are:

- Netrin-1 reduces inflammation by activating A2B-AR which inhibits neutrophil migration.
- SNARE interacts with A2B-AR mainly located within the cell, recruits the receptor to the membrane where a complex with E3KARP, NHERF2, and Ezirin, stabilises A2B-AR on the membrane.
- P105 which, by binding to A2B-AR, inhibits the activity of NF-κB with an anti-inflammatory effect.
- α-actinin-1, which promotes the dimerisation of A2A-AR with A2B-AR, thus inducing the expression of A2B-AR on the cell membrane.

### A3-AR

The A3-AR receptor couples with an inhibitory G-protein isoform Gi3 and like the A1-AR receptor lowers cytosolic cAMP production resulting in the inhibition of PKA with a consequent increase in glycogen synthase kinase-3-beta (GSK-3β) (Table 1 and Fig. 2) [3, 11, 12]. In addition, A3-AR downregulates β-catenin, cyclin D1 (Fig. 2), and c-Myc with inhibitory effects on the cell cycle and also reduces the capability of NF-κB to bind to DNA. A3-AR has also the ability to increase the activity of several kinases, PI3K, p38, ERK1/2, JNK1/2, and PLC (Table 1 and Fig. 2) [3, 11, 12]. This receptor is expressed in numerous tissues and in various cell types including immune cells (Table 1 and Fig. 2), microglia, astrocytes, enteric neurons, epithelial cells, and colon mucosa cells [3, 11, 12].

## Adenosine system in immune cells

**Fig. 3:**
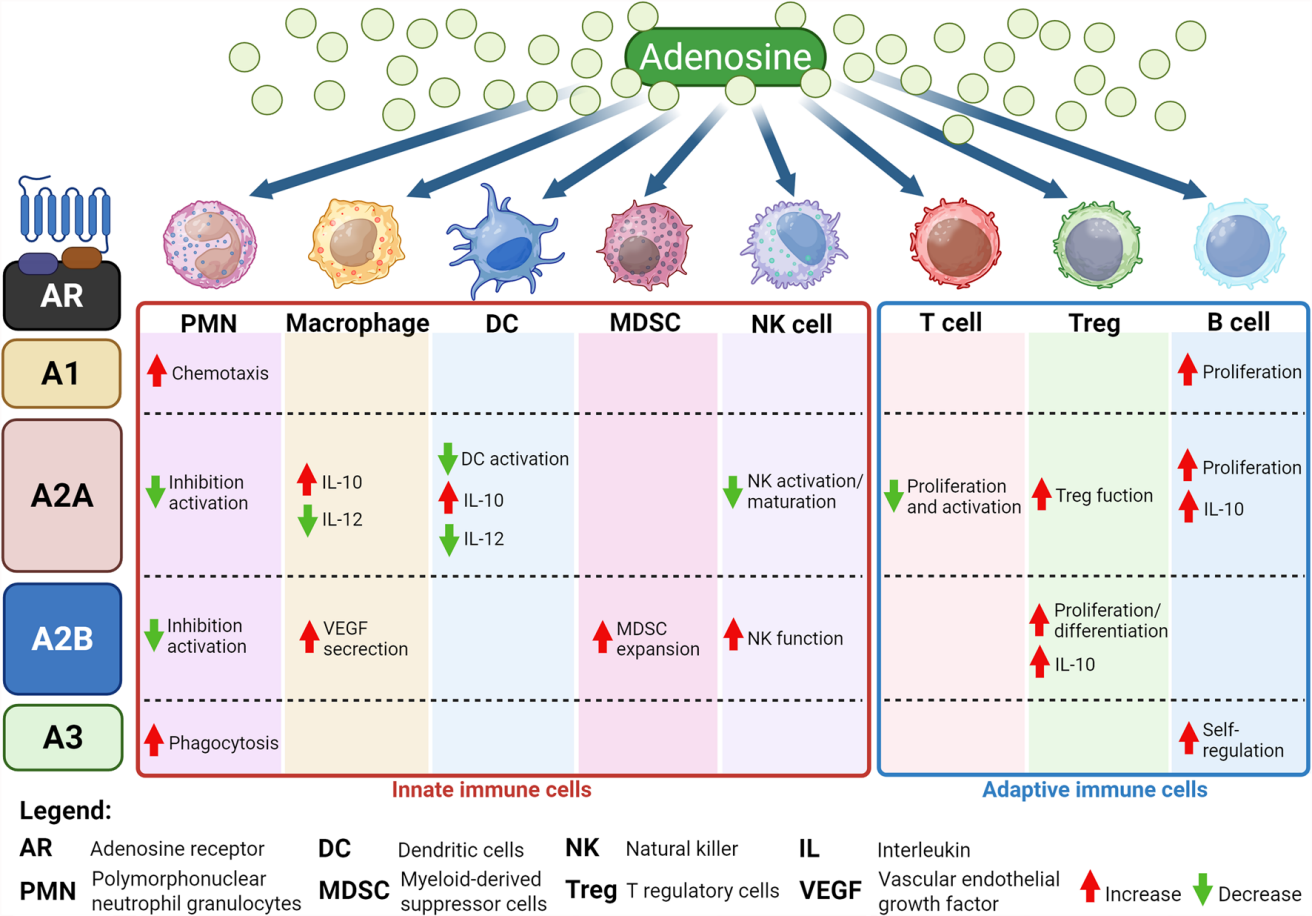
Interaction of Adenosine with Immune Cells: Main actions mediated by ARs expressed by the immune cells. In innate immunity cells: A1-AR is expressed in PMNs, monocyte-macrophages and DCs; A2A-AR is expressed in PMNs, DCs, monocyte-macrophages, MCs and NK; A2B-AR is expressed in PMNs, macrophages, NK, MCs and MDSCs; A3-AR is expressed in PMNs, monocyte-macrophages, DCs and MCs. In adaptive immunity cells: A1-AR is expressed in B lymphocytes, A2A-AR, and A3-AR are expressed in both B lymphocytes and effector T lymphocytes, A2B-AR is expressed in T lymphocytes, whereas Treg lymphocytes express only A2A-AR and A2B-AR. Breg express A1-AR, A2A-AR and A2B-AR. In the Figure are shown the main effects induced by the interaction of ADO with the predominantly express ARs for each cell type. "Created in BioRender https://BioRender.com/oeb1umq"

ADO is a key mediator of the immune response. In fact, ARs are expressed in all types of immune cells (Table 1), where are involved in both the physiology of the immune system and the regulation of immune and inflammatory responses (Table 2 and Fig. 3) [13–16]. ADO through its activation mainly plays a protective role [13–16].

**Table 2:** ARs and Innate Immune Cells: Effects on Cell Functions and on Immune-Mediator Production

Innate Immune cells:	A1-AR		A2A-AR		A2B-AR		A3-AR	
	Effects on cell function	Immune-mediator production	Effects on cell function	Immune-mediator production	Effects on cell function	Immune-mediator production	Effects on cell function	Immune-mediator production
PMN	**↑** Chemotaxis		**↓** Chemotaxis				**↑** Chemotaxis	
	**↑** Adhesion		**↓** Adhesion		**↓** Adhesion		**↓** Adhesion	
	**↑** Phagocytosis		**↓** Phagocytosis		**↓** Phagocytosis		**↑** Phagocytosis	
			**↓** Activation		**↓** Activation		**↓** Activation	
			**↓** Degranulation		**↓** Degranulation		**↓** Degranulation	
	**↑** Antimicrobial activity	**↑** ROS	**↓** Anti-microbial activity	**↓** ROS	**↓** Anti-microbial activity	**↓** ROS	**↓** Anti-microbial activity	**↓** ROS
			**↓** Angiogenesis	**↓** VEGF	**↓** Angiogenesis	**↓** VEGF		
	**↓** Pro-inflammatory cytokines		**↓** Pro-inflammatory cytokines	**↓** IL-8 **↓** TNF-α **↓** IL-6		**↓** TNF-α		
Monocytes- Macrophages	↑ Differentiation						**↑** Chemotaxis	
			**↑** Angiogenesis	**↑** VEGF	**↑** Angiogenesis	**↑** VEGF	**↑** Angiogenesis	**↑** VEGF
			**↑** Anti-Inflammatory cytokines and mediators	**↑** IL-10 **↑** MMP-9	**↑** Anti-Inflammatory cytokines and mediators	**↑** IL-10 **↑** Arginase	**↑** Anti-Inflammatory cytokines	**↑** IL-10
	↓ Pro-inflammatory cytokines	**↓** IL-1β **↓** MIP-1 **↓** MMP-12	**↓** Pro-inflammatory cytokines	**↓**TNF-α **↓** IL-6 **↓** MIP-1	**↓** Pro-inflammatory cytokines	**↓**TNF-α **↓** IL-6 **↓** MIP-1	**↓** Pro-inflammatory cytokines	**↓**TNF-α **↓** IL-6 **↓** MIP-1
			**↓** T cell priming	**↓** IL-2 **↓** IL-12	**↓** T cell priming	**↓** IL-2 **↓** IL-12	**↓** T cell priming	**↓** IL-2 **↓** IL-12
	**↑** Anti-microbial activity	**↑** iNOS **↑** NO	**↓** Anti-microbial activity	**↓** NADPH **↓** ROS **↓** iNOS **↓** NO	**↓** Anti-microbial activity	**↓** iNOS **↓** NO	**↓** Anti-microbial activity	**↓** NADPH **↓** ROS **↓** iNOS **↓** NO
DC	**↑** Chemotaxis				**↑** Chemotaxis		**↑** Chemotaxis	
	**↑** Maturation/ Differentiation						**↑** Maturation/ Differentiation	
			**↓** Activation					
	**↓** Innate Immune Response				**↓** Monocyte differentiation			
	**↑** Angiogenesis	**↑** VEGF			**↑** Angiogenesis	**↑** VEGF	**↑** Angiogenesis	**↑** VEGF
	**↑** Anti-Inflammatory cytokines	**↑** IL-10 **↑** TGF-β	**↑** Anti-Inflammatory cytokines	**↑** IL-10	**↑** Anti-Inflammatory cytokines and mediators	**↑** IL-10 **↑** TGF-β **↑** IDO-1	**↑** Anti-Inflammatory cytokines	**↑** IL-10 **↑** TGF-β
	**↑** Pro-inflammatory cytokines	**↑** IL-6 **↑** IL-8			**↑** Pro-inflammatory cytokines and mediators	**↑**TNF-α **↑** IL-6 **↑** IL-8 **↑** COX-2	**↑** Pro-inflammatory cytokines and mediators	**↑** IL-6 **↑** IL-8 **↑** COX-2
	**↓** Pro-inflammatory cytokines	**↓**TNF-α	**↓** Pro-inflammatory cytokines	**↓**TNF-α	**↓** Pro-inflammatory cytokines	**↓**TNF-α	**↓** Pro-inflammatory cytokines	**↓** TNF-α
	**↓** T cell priming	**↓** IL-2	**↓** T helper 1 priming	**↓** IL-12	**↓** T helper 1 priming	**↓** IL-12	↓ T helper 1 priming	↓ IL-12
					**↓** T helper 2 priming			
					**↓** Cytotoxicity			
					**↑** T helper 17 response			
					**↑** Immune Response	**↑** IFN-γ		
Mast Cells			**↑** Angiogenesis	**↑** VEGF	**↑** Angiogenesis	**↑** VEGF	**↑** Angiogenesis	**↑** VEGF
					**↑** Pro-inflammatory cytokines	**↑** IL-1β **↑** IL-6 **↑** IL-8		
					**↑** Degranulation	**↑** histamine **↑** serotonin **↑** proteases **↑** chemokines	**↑** Degranulation	**↑** histamine **↑** serotonin **↑** proteases **↑** chemokines
					**↑** Anti-Inflammatory cytokines and mediators	**↑** TGF-β **↑** IDO-1	**↑** Anti-Inflammatory cytokines	
			**↑** Cytokines release	**↑** IL-13	**↑** Cytokines release	**↑** IL-4 **↑** IL-13	**↑** Cytokines release	**↑** IL-13
			**↓** Degranulation		**↓** Degranulation		**↓** Degranulation	
			**↓** Cytokines synthesis		**↓** Cytokines synthesis		**↓** Cytokines synthesis	
Natural Killer			**↓** Maturation					
			**↓** Activation					
			**↓** Proliferation					
			**↓** Cytotoxicity		**↑** Cytotoxicity		**↑** Cytotoxicity	
			**↓** Cytokines release	**↓** TNF-α **↓** IFN-γ				

**↑** Increase; **↓** Decrease

### Neutrophils

Polymorphonuclear neutrophil granulocytes (PMN) are characterised by great phenotypic heterogeneity and functional versatility. 

These cells are an important source of ADO, particularly in inflammatory conditions, when they release ATP that is rapidly converted to ADO via CD39 and CD73 expressed right on the membrane of PMNs [3, 13, 16–20]. Moreover, the level of ADO thus generated is maintained by the inflammation-decreased ADO metabolism, ADA deactivation, and decrease of equilibrative transporters.

All four subtypes of ARs are expressed in PMNs (Table 1) [3, 11–13, 16, 17]. In particular, A1-AR promotes the chemotaxis of PMNs, while A2A-AR and A2B-AR inhibit PMN activation (Table 2 and Fig. 3) [3, 13, 17–21]. The inhibitory role of A2A-AR in PMNs is evident when the receptor is stimulated with the agonist CGS21680: in fact, there is a reduction in the phosphorylation of p38, ERK1/2, PI3K/AKT, HCK, and Syk kinase [22]. In addition, the A2A-AR reduces the release of inteleukin-8 (IL-8) resulting in reduced neutrophil degranulation (Table 2 and Fig. 3) [3, 13, 17–19, 21].

PMN phagocytosis is also regulated by ARs; A1-AR increases it, while A2A-AR reduces it (Table 2 and Fig. 3) [3, 13, 17–21]. Activated A3-AR also promotes the formation and rapid extrusion of membrane projections that enhance bacterial phagocytosis and chemotaxis (Table 2 and Fig. 3) [3, 13, 17–19, 21] Finally, ADO has different effects on reactive oxygen species (ROS) depending on the activated AR subtype: activated A1-AR induces ROS production by activated PMNs whereas activation of the A2A-AR receptor downregulates ROS generation (Table 2) [3, 13, 17, 18]. Furthermore, stimulation of A2B-AR and A3-AR suppresses stimulus-induced ROS production in PMNs (Table 2) [3, 13, 17, 18].

### Monocytes-macrophages

ARs are present in both monocytes and macrophages, with different levels of expression depending on the maturation state of the cells [3, 11, 12]. In quiescent monocytes, there is a modest expression of A1-AR, A2A-AR, and A3-AR, which then increases with differentiation into macrophages (Table 1). Inteleukin-1 beta (IL-1β) and Tumour necrosis factor alpha (TNF-α) increase the expression and functionality of A2A-AR in human monocytes, thus preventing receptor desensitisation [3, 11, 12].

The anti-inflammatory effect in macrophages is due to the activation of A2A-AR, A2B-AR, and A3-AR and extracellular ADO, with A2A-AR playing an important role (Table 2 and Fig. 3) [3, 10–12, 17]. The activation of ARs in macrophages has an important role by blocking the release of pro-inflammatory mediators such as TNF-α, inteleukin-6 (IL-6), inteleukin-12 (IL-12), nitric oxide (NO), and macrophage inflammatory protein-1 (MIP-1), while ADO acting on A2A-AR and A2B-AR promotes the release of inteleukin-10 (IL-10) (anti-inflammatory) (Table 2 and Fig. 3) [3, 13, 16, 17, 23–26]. In addition, stimulation of A3-AR promotes chemotaxis of macrophages towards apoptotic cells (Table 2 and Fig. 3) [3, 11, 12, 23].

CD39 and CD73 are critical for the regulation of development, differentiation, and macrophage activity because the ADO they generate can downregulate the expression of the inflammatory cytokines TNF-α, IL-6 and inteleukin-2 (IL-2) and upregulate the expression of the anti-inflammatory ones IL-10 and vascular endothelial growth factor VEGF (Table 2 and Fig. 3) [3, 13, 16, 17, 23–26]. Indeed, CD39 and CD73 are downregulated in pro-inflammatory M1 macrophages and upregulated in anti-inflammatory M2 macrophages, confirming that the balance between ADO and ATP in the microenvironment is essential for inflammation regulation. In addition, A2A-AR increases polarization in M2 macrophages.

### Dendritic cells

Immature Dendritic cells (DCs) mainly express A1-AR and A3-AR (Table 1) by which they regulate chemotaxis through the transient increase of intracellular Ca^2+^, resulting in actin polymerisation, and the increase of co-stimulatory molecules [3, 11–13, 16, 17, 27]. In contrast, in mature DCs, there is a predominant expression of A2A-AR, which after activation causes an increase of cAMP and secretion of IL-10 and a decrease in the pro-inflammatory cytokines IL-12 and TNF-α (Table 2 and Fig. 3**)** [3, 11–13, 16, 17, 27]. However, mature DCs express also A1-AR, A2B-AR and A3AR (Table 1) and effects are reported in Table 2. A2B-AR has a pro-inflammatory effect; indeed, this receptor shifts the differentiation of bone marrow precursors by directing them towards a specific subpopulation of DCs that activate a Th17 cell response (Table 2) [3, 11, 13, 16, 17, 27, 28]. The latter is important in the defence against extracellular pathogens, particularly at the level of the mucosa and epithelial barriers, but its excessive activation is associated with the pathogenesis of various autoimmune diseases.

ADO activates human monocyte-derived DCs via A2A-AR [3, 11–13, 16]. However, unlike normal myeloid DCs, ADO-conditioned DCs express a high level of angiogenic, proinflammatory, immunosuppressive, and tolerogenic factors (Table 2 and Fig. 3). In addition, ADO-conditioned DCs can regulate the polarisation of certain T-cell subtypes (Table 2 and Fig. 3) [3, 11–13, 16, 17, 29, 30]. A2B-AR has also a pro-inflammatory role in DCs because its activation drives them towards differentiation characterised by a pro-angiogenic and pro-inflammatory phenotype. Indeed, under hypoxic conditions, stimulation of A2B-AR causes the release of pro-angiogenic mediators such as IL-6, IL-8, Trasforming Growth Factor beta (TGF-β), VEGF, Indoleamine 2,3-dioxygenase 1 (IDO-1), and cyclooxygenase-2 (COX-2) (Table 2), which in the presence of IL-10 and arginase-2 can induce immunosuppression [3, 10, 11, 13, 16, 17, 27, 30]. In addition, A2B-AR in association with ADA forms a complex with CD26 on the membrane of T lymphocytes, that leads to the release of TNF-α and interferon gamma (IFN-γ) (Table 2) [3, 10–13, 16, 17, 27, 30].

CD39 in DCs can regulate immunological synapses and intracellular signalling. ATP's ability to promote immunosuppression is due to the downregulation of pro-inflammatory cytokines in DCs (Table 2 and Fig. 3) [17, 21, 27, 29–31]. Furthermore, CD39 is necessary for Langerhans cells to achieve an optimal antigen-presenting function [21, 27, 31]. Finally, IL-27 can stimulate CD39 expression in DCs, via STAT3, resulting in repression of the immune response mediated by T helper (Th) 17 (Th17) and Th1 lymphocytes [21, 27, 28, 31]. The expression of CD73 in follicular DCs is essential for the adhesive interaction with B lymphocytes of germinal centres [21, 27, 28, 31, 32].

### Mast cells

AR receptors are expressed in human skin mast cells (MCs) [3, 11–13]. In human LAD2 and HMC-1 MC lines, there is an expression of A2A-AR, A2B-AR, and A3-AR but not of A1-AR (Table 1) [3, 11–13]. In murine MCs, activation of A2B-AR and A3-AR triggers degranulation, leading to the release of histamine, serotonin, chemokines, and proteases (Table 2) [3, 11–13]. Thus, activation of A2B-AR is mainly involved in the degranulation of MCs, while stimulation of A3-AR appears to mediate anti-inflammatory effects (Table 2) [3, 13, 17, 33, 34]. The combination of A2A-AR and A2B-AR activation is necessary for the inhibition of cytokine synthesis in MCs (Table 2) [3, 10, 11, 13, 17, 33, 34]. Based on experiments with KO mice for A2B-AR, MCs release IL-13 and VEGF in response to extracellular ADO suggesting a role of A2B-AR in angiogenesis (Table 2) [3, 10, 11, 13, 35].

### Myeloid-derived stem cells

Myeloid-derived stem cells (MDSC) are a heterogeneous group of immature myeloid cells, i.e. this pool consists of DC, macrophage, and granulocyte progenitors [17, 36]. In fact, the polymorphonucleated MDSCs, called granulocytic MDSCs, morphologically resemble to neutrophils and the monocytic MDSCs morphologically resemble monocytes. The immunosuppressive functions of MDSCs are partially attributable to CD39 and CD73 [3, 13, 17, 21, 37–39]. TGF-β signalling is important for the development of terminally differentiated mononuclear myeloid cells. TGF-β and HIF-1α are involved in the regulation of CD39 and CD73 expression in MDSCs, cells upregulating TNF-β, HIF-1α, TGF-β, COX-2 and IL-10 [3, 13, 17, 21, 37–39]. Inhibition of CD39 and CD73 can alter the inhibitory effect of MDSCs on T cells [3, 13, 17, 21, 37–39].

### Natural killer cells

The expression level of CD73 in Natural killer (NK) cells is negligible [40]. Human NK cells upregulate CD73 expression after contact with mesenchymal stromal cells (MSC) [40]. In addition, human NKs produce ADO in a CD38-mediated manner [41]. Finally, stimulation of A2A-AR in NKs causes an attenuation of immune responses (Table 2 and Fig. 3) while stimulation of A2B-AR and A3-AR decrease cytotoxic activity [3, 11–13, 16, 17].

### Effector T cells

ADO is an important regulator of T lymphocyte functions since through its action on the various AR receptors it can inhibit their mobility, migration, and adhesion as well as to modulate their effector and regulatory functions (Table 3 and Fig. 3) [13–16]*.* After antigen presentation, the subsequent activation of T lymphocytes results in ATP production that stimulates MAPKs. The ATP secreted by activated T lymphocytes and transformed into ADO by ecto-nulceotidases, CD39 and CD73, generate an autocrine and paracrine loop that can promote immunosuppression (Table 3 and Fig. 3) [13–15, 21, 31]. For instance, activation of A2A-AR, which is mainly expressed in T-cells, can inhibit the proliferation and differentiation of naive T-cells and suppress the production of IL-2 and the differentiation in Th1 and Th2 (Table 3 and Fig. 3) [42–44]. In this regulation of effector T cells by ADO, a key role is played by CD39 and CD73 [16, 21, 31, 45–48].

**Table 3:** ARs and Adaptive Immune Cells: Effects on Cell Functions and on Immune-Mediator Production

Adaptive Immune Cells:	A1-AR		A2A-AR		A2B-AR		A3-AR	
	Effects on cell function	Immune-mediator production	Effects on cell function	Immune-mediator production	Effects on cell function	Immune-mediator production	Effects on cell function	Immune-mediator production
T Cells			**↓** Proliferation		**↓** Proliferation		**↓** Proliferation	
			**↓** Adhesion		**↓** Adhesion			
			**↓** Migration		**↓** Migration			
			**↓** Activation	**↓** NF-κB **↓** IL-2	**↓** Activation	**↓** NF-κB **↓** IL-2	**↓** Activation	**↓** NF-κB **↓** IL-2
			**↓** Pro-Inflammatory	**↓** TNF-α **↓** IL-1β **↓** IL-6 **↓** MMP-1 **↓** MMP-3				
			**↓** T helper naive and T helper 1 response	**↓** IFN-γ	**↓** T helper naive and T helper 1 response	**↓** IFN-γ	**↓** T helper 1	**↓** IFN-γ
			**↓** T helper naïve and T helper 2 response	**↓** IL-4				
			**↓** T helper 17 response		**↑** T helper 17 response			
			**↓** Cytotoxicity	**↓** exocytosis granules **↓** Fas Ligand	**↓** Cytotoxicity	**↓** exocytosis granules **↓** Fas Ligand		
Treg Cells	**↑** Proliferation		**↑** Proliferation		**↑** Proliferation			
	**↑** Differentiation		**↑** Differentiation		**↑** Differentiation			
	**↑** Functions		**↑** Functions		**↑** Functions			
	**↑** Anti-Inflammatory	**↑** IL-10 **↑** Foxp3	**↑** Anti-Inflammatory	**↑** IL-10 **↑** Foxp3	**↑** Anti-Inflammatory	**↑** IL-10 **↑** Foxp3		
	**↓** Pro-Inflammatory release by effector T cells	**↓** NF-κB	**↓** Pro-Inflammatory release by effector T cells	**↓** NF-κB	**↓** Pro-Inflammatory release by effector T cells	**↓** NF-κB		
B Cells	**↑** Proliferation		**↑** Proliferation				**↓** Proliferation	
	**↑** Differentiation Plasma cells		**↑** Differentiation Plasma cells					
	**↑** Isotype switch Plasma cells		**↑** Isotype switch Plasma cells					
			**↑** Anti-Inflammatory	**↑** IL-10				
	**↑** Cytokine expression						**↓** Cytokine expression	
	**↑** Proliferation of T cells by resting B cells							
	**↓** Proliferation of T cells by activated B cells		**↓** Proliferation of T cells by activated B cells				**↓** Proliferation of T cells by activated B cells	
	**↓** Cytokine expression and Function of T cells dependent on B cell activation state and microenvironment						**↓** Cytokine expression by T cells	
							**↑** Self-regulation	
Breg Cells	**↑** Proliferation		**↑** Proliferation					
	**↑** Functions		**↑** Functions					
			**↑** Regulation of T cells					
			**↑** Anti-Inflammatory	**↑** IL-10	**↑** Anti-Inflammatory	**↑** IL-10		

**↑** Increase; **↓** Decrease

CD39 is mainly expressed in CD4 + and CD39 + T lymphocytes (CD4 + CD39 +), which are the most sensitive to apoptosis and to metabolic stress [21, 31, 32, 45–47]. Indeed, CD39 expression in activated T lymphocytes is associated with lymphocyte reduction. Furthermore, CD39 is also expressed in CD8 + T lymphocytes (CD8 + CD39 +), which mainly mediate specific killer activity, but also have NK-like activity [31, 47]. Various stimulation events of CD8 + T lymphocytes regulate the expression of CD39: in fact, their stimulation via CD3/CD28 induces the production of ROS and the expression of CD39. In addition, inhibition of nicotinamide adenine dinucleotide phosphate hydrogen (NADPH) oxidase also upregulates the level of CD39 [49]. Further, both tumour growth factor-β (TGF-β) and IL-6 are involved in the upregulation of CD39. In T lymphocytes CD8 + CD39 + the secretion of IL-2, TNF-α, IFN-γ is decreased while the expression of immune checkpoint inhibitors such as lymphocyte activation gene 3 (LAG-3), programmed cell death protein1 (PD1), T cell Ig and iTIM domain (TIGIT), T cell Ig mucin domain-3 (TIM-3) is upregulated [21, 31, 45, 46, 50–52]. An upregulated level of this CD8 + CD39 + T lymphocyte population indicates that a progressive depletion of the T-lymphocyte population is maturing [21, 53].

CD73 is only expressed in a small percentage of CD4 + T lymphocytes but increases in this population in inflammatory states [21, 31, 32, 45–48]. TNF-α, TGF-β, retinoic acid and the active form of vitamin D upregulate CD73 expression in both CD4 + T lymphocytes and CD8 + T lymphocytes [21, 31, 32, 45–48]. Furthermore, peripheral CD8 + T lymphocytes that have developed predominantly from naive T-lymphocytes can also express CD73 [14, 31, 32, 48]. Downregulation of CD73 in CD8 + T lymphocytes can interrupt autocrine ADO signalling and promote their differentiation [14, 31, 32, 48]. ADO derived from CD73 + T lymphocytes stimulating A2A-AR can regulate effector cell differentiation by suppressing the WNT pathway [54, 55]. Actually, WNT signalling can prevent effector T lymphocyte differentiation and maintain the stemness of CD8 + T lymphocyte memory cells [56].

Th17 is a subset of T-cells that produce IL-17, with which they regulate inflammation. They also produce GM-CSF and IFN-γ. TGF-β and IL-6 upregulate STAT3 and downregulate Gfi-1, which are crucial for inducing the expression of CD39 and CD73 in Th17 [16, 21, 31, 32, 45–48, 57, 58]. This, therefore, enables Th17s to produce ADO resulting in the suppression of immune responses (Table 3 and Fig. 3) [13–16, 59] Despite this, Th17s remain capable of fostering anti-cancer immune responses. The expression of CD39 and CD73 also leads to downregulation of granzyme B and IFN-γ [16, 21, 31, 32, 45–48, 60]. Finally, ATP via P2X7R activation promotes Th17 recruitment [57, 58].

### Regulatory T cells

The canonical ADO pathway plays an important role in the regulation of immune responses [13–15, 31]. CD25 + FOXP3 + regulatory T-cells (Treg) express CD73 and are capable of producing ADO from the AMP present at the inflammatory site, which serves to suppress T-lymphocyte proliferation and cytokine secretion by reducing excessive immune responses (Table 3 and Fig. 3) [17, 21, 31, 46, 59, 60]. CD39 is also involved in regulation being expressed in all CD4 + CD25 + T cells which in humans identifies a subset of Treg involved in the control of autoimmune inflammatory disease [50].

Co-expression of CD39 and CD73 is a peculiarity of Treg compared to other cell subsets [14, 16, 17, 32, 45–47, 59–61]. These cells, which are capable of producing ADO via the canonical pathway, inhibit effector functions mainly through the interaction of ADO with A2A-AR (Table 3 and Fig. 3), thus promoting immunosuppression and upregulation of PD-1 and CTLA-4 [59, 60, 62]. Furthermore, A2A-AR activation induces Treg expansion resulting in further immunosuppression with a self-reinforcing loop (Table 3 and Fig. 3) [17, 59–62]. Finally, activation of A2-AR reduces the release of pro-inflammatory cytokines through activation of NF-κB, also contributing to the suppression of immune responses is the interaction between Treg and DCs that actively produces ADO (Table 3 and Fig. 3) [59, 61, 62].

### B lymphocytes

B lymphocytes express all the main components of the adenosine system: ecto-nucleotidase, deaminase, kinase, nucleotide transporters, and ARs, excluding A2B-AR, so are capable of producing ADO and regulating its levels (Table 1) [2, 3, 10, 11, 13–16]. ADO is involved in regulating the development, implantation, and maintenance of the plasma cell population in the bone marrow during the primary immune response and in the management of Ig class switching by regulating the recombination events (Table 3) [63–65]. This is because CD39 and CD73 in B lymphocytes can perform an autocrine ADO loop. The regulatory role of ADO in the functionality of B lymphocytes is clearly demonstrated by the fact that in inactivated B cells there is a higher extracellular concentration of ADO and when activated their ATP release increases, reducing the inhibitory effects induced by ADO [63–65]. Thus, activated B cells protect their activated state and exert a pro-inflammatory role after activation [2, 3, 11–14] In addition, ADO can regulate the functions of regulatory B lymphocytes (Breg), a subset of immunosuppressive cells involved in immune tolerance (Table 3 and Fig. 3) [63, 66].

The co-expression of CD39 and CD73 in resting circulating B lymphocytes suggests that they can hydrolyse ATP and thus produce AMP and ADO [64]. The expression level of CD39 and its enzymatic activity in B lymphocytes may be upregulated by CD40L and inteleukin-4 (IL-4) [63, 64, 66]. Moreover, ADO is mainly destroyed by the same B-cells that produce it, for the self-regulation of their function via the A3-AR (Table 3 and Fig. 3) [3, 11, 12].

Breg regulates not only their own function but also the activities of T lymphocytes through ADO signalling which originates from the enzymatic degradation of ATP released in the extracellular space by activated immune cells (Table 3 and Fig. 3) [2, 3, 13, 14, 63, 66]. In humans, B-cells activated by downregulation of CD73 and upregulation of CD39 can inhibit T-lymphocyte-mediated immune responses (Table 3 and Fig. 3) [63, 66]. Since CD39 + CD73 + B cells can produce AMP and ADO, they can regulate the immune responses of CD4 + T lymphocytes and CD8 + T lymphocytes (Table 3 and Fig. 3) [63, 66]. In addition, Breg via CD39 and IL-10 can repress T-lymphocyte-mediated responses (Table 3 and Fig. 3) [66]. Resting B lymphocytes, however, can promote the proliferation of activated T lymphocytes through the production of cytokines, whereas ADO is degraded by ADA expressed on T lymphocytes (Table 3 and Fig. 3) [63–65]. The functional regulation of Breg is due to A1-AR and A2A-AR that contribute to the proliferation of CD39 + B lymphocytes, while IL-10 production is due to the activation of A2A-AR (Table 3 and Fig. 3) [63, 66]. B lymphocytes are also involved in the generation of CD39 + CD73 + extracellular vesicles that can suppress the anti-tumour immune response [67].

## Adenosine and ATP dialogue

Under physiological conditions, the extracellular concentration of ATP and ADO is very low, with an increase after necrosis and apoptosis [1–4, 15]. In fact, the concentration of ATP is 400–1000 nM and can increase three or more times, while the concentration of ADO, which is 40–80 nM, can reach values of 100–500 nM after necrosis and 100–200 nM after apoptosis.

In the extracellular space, nucleotides and purinergic nucleosides exert their effects through interaction with specific membrane receptors, called purinergic receptors (purinoreceptors). They are divided into two groups, the P1R group, which has ADO as its endogenous agonist, and the P2R group, which is sensitive to di- and tri-phosphate nucleosides (ATP, ADP, UTP, UDP) [11]. ATP, when it becomes extracellular (eATP), acts as a danger-associated molecular pattern (DAMP), binding to P2 receptors and thus initiating the cascade that induces an inflammatory response [14, 16, 68–70].

An important mechanism for avoiding the pathological effects of ATP is its hydrolysis to ADO by C balance near to the cell and the types of ARs expressed in the cells [1–6, 15].

ATP is actively released by activated or stressed cells during events such as inflammation, hypoxia, and apoptosis [14, 70–75]. Passive release, on the other hand, occurs from necrotic cells by rupturing of the plasma membrane. Activated or apoptotic cell release occurs mainly by two mechanisms, namely exocytosis of intracellular vesicles, by neuronal cells, lymphocytes, endothelial cells, or transport via membrane-bound channels or transporters. These two mechanisms can act together in the same cell. Cells that produce exosomes containing ATP in intracellular vesicles release them into the extracellular environment by a mechanism of vesicular exocytosis, whereby the vesicles are incorporated into the plasma membrane and then released into the extracellular environment [76]. The conductive release of ATP from cells is associated with two types of plasma membrane channels [77, 78]: the first consists of Cl^−^ channels, such as maxi-ion channels present in endothelial cells and various types of immune cells. These channels allow the passage of small organic anions such as ATP and are activated by osmotic swelling and during hypoxia. The second type consists of volume-regulated ion channels or pore-forming channels, such as connexins or pannexins. They are permeable to organic anions such as ATP and are activated by osmotic swelling. Such channels are present in endothelial cells and macrophages [77, 78].

Connexins are molecules forming part of the structures that contribute to the molecular gap-junction complex. Further, they also form connexin channels in non-junctional regions of the plasma membrane that allow molecules to pass into the extracellular environment. Connexin channels respond to membrane depolarisation or lowering of the extracellular Ca^2+^ concentration. Certain pro-inflammatory stimuli also induce the release of ATP by connexins channels. Finally, certain types of channels as Connexins 43 or Connexins 32 are involved in the extracellular release of ATP [77–79].

### ATP system

The ATP dephosphorylation system is characterised by three key events [80]:

1. CD39 dephosphorylate ATP to ADP to AMP
2. Alkaline phosphatase (AP) dephosphorylate ATP to ADP to AMP
3. CD73 dephosphorylate AMP to ADO

Furthermore, the bioavailability of extracellular ADO is regulated by ADA, which converts it to inosine, and by transport into the cell by nucleoside transporters.

In the extracellular compartment, ATP binds to purinergic P2 receptors, divided into two subsets [11, 14, 71, 73, 81]: (a) P2X subset containing: P2X1-P2X7 (in humans) have a common molecular organisation consisting of two transmembrane domains, a large extracellular loop, and two carboxyl and NH_2_ intracellular ends; the NH_2_ end contains a consensus site for PKC phosphorylation indicating that the phosphorylation state of P2X subunits may be involved in receptor function; the majority of subunits can form functional monomeric, homomeric or heteromeric receptors. They are plasma membrane channels activated solely by ATP to mediate the influx or efflux of various cations, Na^+^, K^+^ and Ca^2+^ [11, 71, 82, 83]**.** In humans, the seven P2X receptor subunits arranged as trimers with 3 receptors located around ion-permeable channels After binding of three ATP molecules, the subunits rearrange and the ion channels open, resulting in ion fluxes and membrane depolarisation and activation of signalling cascades, such as MAPKs [11, 71, 82, 83]. P2X receptors are involved in numerous physiological processes, including macrophage activation and induction of apoptosis, and are expressed in various cell types including lymphocytes, macrophages, and glial cells [11, 14, 71, 73, 81, 84].

b) P2Y subset, which consists of G-protein-coupled receptors that modulate signalling events such as activation of AC, PLC, and ion channels activation. This subset contains P2Y1-P2Y14 (there are only 8 of them), predominantly activated by ATP or ADP, but there are variations such as for the P2Y2 receptor activated by uridine-5′-triphosphate (UTP).

ATP has a variety of inflammatory effects since immune and endothelial cells express most of its receptors. Due to its ability to regulate inflammatory responses, it is regarded as a damage-associated molecular pattern (DAMP), an endogenous signal that derives from tissues and initiates and regulates immune responses in cooperation with other signals. Its effects stem from ATP's involvement in the main aspects of inflammation: the chemotaxis of inflammatory cells, the production of O_2_ radicals by neutrophils, and the production of cytokines by inflammatory cells. Pro-inflammatory effects are induced when ATP reaches high extracellular levels, which characterise the early stages of inflammation [14, 70–75]. In addition, ATP can activate the inflammasome by binding to P2X7 receptors, which then recruit the pannexin-1 membrane pore that allows agonists to penetrate the cell and thus activate the inflammasome, NLRP3. This is a multi-protein complex that induces caspase-1 activation, also resulting in the secretion of IL-1β and IL-18. It is also possible that ATP, behaving like a DAMP, induces the production of ROS, which then become the main contributors to the formation of the NLRP3 inflammasome [85–89]. ATP also has anti-inflammatory effects, that can appear at low ATP levels, or following chronic exposure to ATP [16, 71, 90]. Another anti-inflammatory mechanism is to prompt angiogenesis and wound repair by inducing VEGF production by monocytes [91].

In general, ADO has opposite effects on inflammation compared to ATP [13, 68]. In fact, it inhibits the adhesion of inflammatory cells to endothelial cells, reduces the release of pro-inflammatory cytokines, promotes the release of the anti-inflammatory cytokine IL-10 by monocytes, and induces the production of VEGF, which is a powerful inducer of angiogenesis and vascular permeability [2–5, 13, 16, 68, 70, 92, 93]. Most of these effects are regulated by the A2-AR receptor [3, 13]. Thus, the dialogue between ATP and ADO (balance) in inflammation can be summarised as follows [2–5, 13, 16, 68, 70, 92, 93]: during inflammation, high levels of ATP are produced by activated, damaged or dying cells at the inflammation site. ATP attracts immune cells to the inflammatory site and activates or increases the activation of the recruited cells, which begin to produce pro-inflammatory factors such as ROS and cytokines that inhibit ATP-dephosphorylating enzymes such as CD39 and CD73, favouring the persistence of inflammation [31, 46]. ATP levels may decrease with cell death and the dephosphorylation of ATP to ADO, which causes a progressive increase of ADO levels. ATP dephosphorylation is facilitated by hypoxia, which increases CD39 and CD73 activity, and decreases activity of intracellular transport by NT so that extracellular ADO remains high. Thus, ADO with its anti-inflammatory properties facilitates the resolution of ongoing inflammation or the inhibition of the onset of inflammation [13, 68].

### ATP and specific immune responses

ATP predominantly stimulates specific immune responses by acting on T lymphocytes, boosting their activation by amplifying T cell receptor (TCR)-induced activation and increasing IL-2 production [47, 94] In addition, ATP induces differentiation, activity, and functions of Treg, and simultaneously stimulates the differentiation of pro-inflammatory Th17 expressing CD39 and/or CD73 by which it decreases local ATP levels by increasing local ADO levels [47, 94–97].

ATP control over immune cells is also regulated by its concentration. At low concentrations, it activates T lymphocytes, while at high concentrations the prolonged stimulation of the P2X7 receptor induces pore formation in the membrane and apoptosis of T lymphocytes [94, 97–100].

ADO exerts its inhibitory function through opposite effects to those of ATP [13–16, 21, 31], with an action characterised by inhibition of T-lymphocyte responses, such as cytotoxicity and cytokine production, inhibition of Treg and Th17 [32, 45–48, 57–60]. It also inhibits the differentiation of Th1 and Th2 lymphocytes by decreasing T-lymphocyte proliferation and IL-2 production [42–44]. ADO also causes the blockade of TCR signalling after inducing an increase in cAMP as a consequence of A2A-AR stimulation. The latter can also inhibit the signalling of NF-κB [59, 61, 62].

In conclusion, ATP activates T lymphocytes by inducing IL-2 and cytotoxicity, and drives differentiation towards pro-inflammatory Th17, while inhibiting differentiation towards Treg. Instead, ADO inhibits IL-2 production and differentiation towards Th17 while stimulating differentiation towards Treg.

## The intestinal mucosal system

### Characteristics and functionality of intestinal epithelia

Intestinal epithelia (IE) is made up of a single layer of cells acting as a physical barrier, and is crucial for the preservation of intestinal homeostasis [101, 102] IE is like a hub that coordinates the immune system defence by regulating the cross-talk between bacteria and immune cells with a complex communication process, playing a central role in the enteric defence of the host [103]. Distinct sets of intestinal epithelial cells (IECs) promote homeostasis using specific immune mechanisms [101–104]. The IE extends over the villi that protrude into the intestinal lumen, thereby increasing the surface area of the mucosa and the absorption of nutrients [104]. The flat surface of the epithelium is characterised by Lieberkühn’s crypts that appear as invaginations [104]. At the base of the crypts, there are intestinal stem cells (ISCs) that give rise to cells with transient proliferative activity, which differentiate and mature while crossing the transition zone [104, 105]. IECs are distributed throughout the lumen of the small intestine crypts and progressively replace dying IECs. Under homeostatic conditions, the entire crypt is renewed every 4–5 days [104]. Various types of differentiated cells are present in the epithelium of the intestine each with unique and specialised functions. They have a different distribution between the small and large intestine [104, 106, 107].

### The cells of the small intestinal epithelium

In the epithelium of the small intestine, the most numerous cells are the enterocytes, mainly located in the crypt-villus axis [104, 106, 107]. Enterocytes are responsible for the absorption of nutrient molecules and water and also secrete the antimicrobial peptides REGIII, β- and γ-defensins, and cathelicidin [104, 106] Paneth cells, on the other hand, are localised at the base of the crypts and produce specific antimicrobial peptides with which they also protect the stem cells underlying the small intestinal crypts, i.e. lysozyme, α-defensins and secreted phospholipase A2 (sPLA2) [104, 106–108]. Goblet cells secrete mucus, also facilitating luminal transfer of antigen to dendritic cells via goblet cell-associated antigen passages (GAP) [104, 106, 107]. Enteroendocrine cells secrete hormones and chemosensory Tuft cells are an important defence against helminths [104, 106, 107]. M-cells continuously capture and present luminal antigens to the immune system. M-cells are localised in the follicle-associated epithelium (FAE), located above the Peyer's plaques [104, 106, 107].

### Colonic epithelial cells

The colonic epithelium is composed of colonocytes (enterocytes), enteroendocrine cells, chemosensory Tuft cells, and Goblet cells that form the outer and inner mucus layer by secreting the mucin, secreted gel-forming mucin 2 (Muc2) [104, 106, 107, 109, 110]. In addition, they secrete the barrier-associated proteins AGR2, 2G16, CCLA1, and RELM-β [106, 111]. In the colon epithelium, Paneth cells and M-cells are missing. Within each type of IEC, the cells have such specialised functions that they are classified into further subsets [104, 106, 107]. These cell subsets are: enterochromaffin cells (5-ht/serotonin), D cells (somatostatin), G cells (gastrin), K cells, I cells, S cells, and further types [103, 104, 106, 109, 112]. The Goblet cells with sentinel Goblet cells, localized at the top of the colon crypts, sense nearby microbes by triggering a rapid release of mucin to ward off harmful stimuli [104, 106, 107, 110]. The Tuft cells, which develop differently depending on whether they are in the small intestine or the colon, form two subsets, Tuft1 expressing the epithelial cytokine TSLP and Tuft2 expressing CD45 [113–115]. Enterocytes differentiate when they migrate at the top along the axis of the crypt [104, 106, 107]. These cells, when in the apical tips, metabolise microbial SCFAs and consume O_2_ in the colon [107, 116–118]. In contrast, the enterocytes at the base of the crypts ferment glucose and lactate and do not consume O_2_ [117, 118]. Paneth cells, on the other hand, have no identifiable subset, and M cells have a subset identified as inflammation-induced M cells [104].

## Intestine and the adenosine system

In the complex regulation and protection of the intestinal epithelial barrier, ADO plays a very important role by contributing to intestinal homeostasis [1, 68, 92, 119–121] and protective effects in intestinal infection and inflammation [119, 122–124]. The ADO system is mainly characterised by the following events: the first event is the production of ADO, the various mechanisms of which we have outlined, the second event is the modulation of the levels of ADO produced, the third event is the receptors for ADO involved, and the fourth event is the signalling pathways activated after the binding of ADO to its receptors [1–5, 12, 13, 73, 92].

The surface of the intestinal epithelium is covered with a layer of mucus that acts as a first line of protection. This layer is single and loose in the small intestine and double in the colon. The production and maintenance of the mucus layer is due to the Goblet cells that secrete mucins. In the colon, the main mucin is the Muc2 [125]. Prior to secretion, Muc2 is stabilised by sialylation by STG sialyltransferase, which allows Muc2 to resist degradation by bacterial enzymes [126]. ADO regulates the mechanisms of mucus production and its secretion [119, 127–130].

The microbiome that colonises the outer mucous layer of the colon and, in addition to resistance to colonisation against pathogenic bacteria, contributes to strengthening the integrity of the barrier [131–133]. The gut microbiota release ATP that has direct antimicrobial effect [134] and that can be converted to ADO, which contribute in indirect manner to various antimicrobial and bacteriostatic effect on various intestinal pathogens [119, 132, 135–138].

The para-cellular space between adjacent intestinal epithelial cells is strongly sealed by the junctional complexes located at the apical regions of the lateral membranes of the cells and consists of tight junctions (TJs), adherent junctions, and desmosomes. TJs are the most critical structures for the integrity of the intestinal epithelial barrier [139–141].

ADO is responsible for preserving the integrity of the barrier in both homeostasis and disease [2, 3, 5, 13, 92, 119–121, 138, 142]. During inflammation, infection, and hypoxia, ADO signalling is responsible for the initiation of repair pathways and for TJ regeneration [3, 5, 13, 92, 93, 110, 119, 143–145]. Thus, the local production of ADO by CD39 and CD73 is important for the regeneration of the intact intestinal barrier, and these two ecto-enzymes are crucial for maintaining the integrity of the intestinal epithelial barrier with the involvement of A2A-AR [21, 31, 32, 48, 119, 146, 147]

The role of ADO in TJ integrity involves mechanisms such as its activity in relation to intestinal mucus layers, the role of ZO-1, capable of interacting with E-cadherin and intestinal chlorius secretion [119–124].

### Other roles of ADO in gut physiology

(A) intestinal Cl^−^ secretion. Cl^−^ acts as a key determinant of the hydration of the mucus layer and its balance [119, 148–150]. In the colon, Cl^−^ efflux occurs via the cystic fibrosis transmembrane conductance regulator (CFTR) or via Ca^2+^-dependent Cl^−^ channels. When Cl^−^ ions leave the cell, they create an osmotic gradient that promotes the movement of water into the lumen to hydrate the mucus layer [119, 148–150]. At this interfacial level, ADO promotes the secretion of Cl^−^ into the intestinal epithelial cells by signalling through A2B-AR thus initiating cAMP-dependent Cl^−^ secretion [119, 148, 151, 152].

(B) restoration of the intestinal acid–base balance [119]. Acidification is a characteristic of intestinal inflammation [119, 153, 154]. The shift towards an acidic pH is due to the accumulation of immune cells at the inflamed site and increased lactate release by intestinal epithelial cells [119, 153, 154] The ability to maintain pH homeostasis requires the secretion of bicarbonate (HCO3^−^) into the lumen. The most important promoter of this secretion is the anion transporter, SLC26A3, which is a key regulator of acid–base homeostasis by facilitating Cl^−^ uptake and HCO3^−^ secretion [119, 154]. ADO is involved in this regulation because it induces the expression of SLC26A3 via the cAMP-CREB pathway, thus limiting intestinal acidification [119, 154].

ADO transporters contribute to the regulation of intestinal barrier function, helping to maintain/regulate ADO levels at the surface epithelium. In fact, ENT1 and ENT2 are expressed in the gut, but there are still no in-depth studies on their role [119, 124, 155]. Thus, ADO displays a protective role toward the main factors regulating colon physiology, namely the microbiota, mucus production, TJs structuring, Cl^−^ secretion, and intestinal pH [119]. Of course, the entire ADO system is involved, which includes receptors, ADO-producing enzymes, and ADO transporters [2–5, 12, 13, 92]. This ADO system modulates the duration and magnitude of the ADO response in the extracellular environment to reinforce the integrity of the intestinal barrier [119, 121, 156]

## Pannexin (Panx) and the P2X7R system

### Panx

Panx is a family of proteins consisting of three members, Panx1-3 that form predominantly large transmembrane channels connecting intra- and extracellular space which are present in many cell types, including immune cells [77–79] through which ions and small molecules such as ATP pass, thus acting as a conduit in response to physiological and pathological (hypoxia or apoptosis) stimuli [157, 158]. These non-junctional transmembrane channels, which allow the transport of molecules below 1000 kDa, are present not only in the membrane but also in the endoplasmic reticulum (ER) and Golgi membranes [157, 158]. In addition to ATP, Panx also transports inositol triphosphate and other small molecules and ions such as Ca^2+^, and can form hemichannels [157–159]. The mechanism underlying Panx-mediated ATP release may involve an increase in Ca^2+^ released by the ER [157–159]. Panx1 is activated by ATP binding to P2X7R [160–163]; this receptor is part of the ATP-gated P2X receptor cation channels family, a family of proteins consisting of cation-permeable ligand-gated ion channels that open in response to ATP [157–159, 164, 165]. The release of ATP by Panx channels also activates purinergic P2Y receptors that induce the formation of inositol-3-phosphate and an increase in intracellular Ca^2+^, promoting the further opening of Panx channels and the propagation of a Ca^2+^ wave through the tissue [157–159, 165–167].

From a structural point of view, Panx consists of four transmembrane domains, 2 extracellular loops, 1 intracellular loop, and 1 intracellular code-N and -C terminals [157, 158, 168]

In macrophages, Panx1 and its P2X7 receptor form a complex signalling system and its activation results in large pore formation, ATP release, and paracrine activity [157, 158] In addition, Panx1 may be involved in cell death when activation of the Panx1/P2X7 complex is prolonged [160–163, 169–171]. In macrophages, it plays an important role in inflammation [157, 158, 167, 172]. Panx1 is also involved in immune functions, T-lymphocyte activation and several of their functional responses [77–79, 157, 158, 173].

Panx1 plays an important role in mediating gut function and in the pathophysiology of Inflammatory Bowel Disease (IBD). In a mouse model of colitis, Panx1 was required for P2X7 receptor-mediated enteric neuron cell death during gut inflammation [79, 171]

Panxs may be involved in the process of tumour development: truncating mutations of Panx1 promote the metastasis of colon-rectal carcinoma (CRC), allowing tumour cells to survive mechanical stress in the microcirculation by releasing ATP [174–177].

### The P2X7R System

The P2X7R receptor belongs to the purine receptor family for ATP and is expressed in the central nervous system, microglia, macrophages, and other tissues [14, 70, 71, 73, 81, 178]. P2X7R functions as a pattern recognition receptor (PRR) for extracellular ATP that mediates the events of apoptosis, regulation of receptor trafficking, MC degranulation, and inflammation, where it induces the NLRP3 inflammasome that then leads to the release of IL-1β [70, 79, 84, 178–181]. P2X7 subunits can only form homeric receptors with a typical P2X receptor structure. The P2X7 receptor is a ligand-gated cation channel that opens in response to ATP binding and results in cell depolarization and requires higher levels of ATP for its activation compared to other P2X receptors [11, 71, 82, 83, 178, 182]. Activation of P2X7R by ATP results in the recruitment of Panx pores that allow small molecules such as ATP to be released from cells [88, 183, 184]. This allows the further activation of purinergic receptors and physiological responses such as the diffusion of cytoplasmic Ca^2+^ waves [88, 178, 183, 184]. This event may be responsible for ATP-dependent lysis of macrophages through the formation of membrane pores permeable to larger molecules [71, 73, 81, 185]. More specifically, the first step in the P2X7R activation process is the binding of ATP at the three subunit interfaces. After it has bound to the extracellular loop of P2X7R, it induces a conformational change in the ion channel structure that causes the ion-permeable pore to open. Cation entry causes membrane depolarization and the activation of various Ca^2+^-dependent intracellular processes. The opening time of the channel depends on the subunits that make up the receptor with some desensitizing rapidly if there is a continuous presence of ATP, while others remain open as long as ATP remains bound. Thus, the molecules involved in the flow can exit or enter in a finely tuned flow equilibrium [71, 81, 88, 178, 181–185].

P2X7R are synthesized in the ER and after a complex glycosylation in the Golgi apparatus are transported to the membrane where docking is achieved via specific members of the SNARE protein family. Removal from the membrane of P2X7Rs occurs by clathrin-mediated endocytosis of the receptors into endosomes where they form vesicles for degradation or recycling [186]. The sensitivity of P2X7Rs to ATP is strongly modulated by changes in extracellular pH and the presence of heavy metals such as zinc and cadmium [11, 178, 181, 182].

## *C. difficile* infection

### *C. difficile* epidemiology and pathogenesis

In intestinal infections caused by toxin-producing bacteria, the ADO system [1, 68, 92, 119–124] consisting of various mechanisms of synthesis of ADO, of regulation of its levels, and its four ARs, is profoundly involved with effects ranging from the regulation of the inflammatory response to the opposite exacerbation of that response [68, 92, 119, 187–189]. This is also a consequence of the fact that inflammatory immune cells and other cell types that make up the heterogeneity of the colon mucosa also express ARs to varying degrees [3, 11, 12, 119, 190].

Adding to this complex picture is also the fact that bacterial toxins, as well as other components of the bacterial cell, can influence the expression and function of ARs [3, 11, 12, 119, 187–191].

In the context of intestinal bacterial infections, an increasingly important role is due to those caused by *C. difficile*, an opportunistic, Gram-positive, anaerobic, and spore-forming bacterium [192, 193]. This bacterium has become the most common cause of antibiotic-associated diarrhea in hospitalized patients [194–196]. The epidemiology of *C. difficile* infection (CDI) has changed over the past two decades [197, 198]. Most cases of CDI were previously correlated to healthcare (HA)-CDI exposure [199–201]; however, recent studies have suggested an increased incidence in community-acquired (CA)-CDI reaching up to 40% of all CDI cases [201–203]. Interestingly, the incidence of multiple recurrent CDI (rCDI) has risen disproportionately to the incidence of CDI [204, 205].

The main reason for the ongoing CDI epidemic is the emergence of the newer, more hypervirulent, antibiotic-resistant, epidemic strain of *C. difficile*, known by the polymerase chain reaction as ribotype 027, North American pulse-field type 1 (NAP1), restriction endonuclease analysis (REA) type B1 strain, or simply “ribotype 027” [206, 207]. In fact, ribotype 027 is associated with more frequent and severe illness which is refractory to antibiotic therapy and has a greater risk of relapse [198, 207, 208]. The emergence of hyper-virulent strains of *C. difficile* and the resistance of the spore in the external environment has favoured its progressive diffusion in the anthropized environment to become ubiquitous. This process of endemization has allowed the progressive colonization of the human gastrointestinal tract making *C. difficile* a considerable threat to public health globally [208, 209].

The pathological manifestations of CDI range from moderate diarrhea to severe colitis, pseudomembranous colitis, and toxic megacolon, resulting in a high mortality rate when perforation with septic shock occurs [194, 196, 208, 210]. The pathological manifestations of CDI are mainly due to the production by *C. difficile* of two Tcds, TcdA and TcdB [210–214]. However, *C. difficile* produces also a binary toxin called *C. difficile* transferase (CDT), with a minor pathogenic contribution [210]. Therefore, TcdA and TcdB are the main responsible for the pathogenic activity of *C. difficile*, with TcdB, being more toxic than TcdA, considered responsible for most pathological effects of CDI [210–212]. Tcds, after binding to receptors on target cells and the following internalization, induce in all cell types examined inactivation of the Rho-GTPase by glucosylation causing cytopathic and cytotoxic effects which leads to the loss of many important biological functions [210–214]. However, Tcds can cause also cytotoxic effects that are glucosylation-independent [210–214]. Moreover, Tcds cause the production/secretion of chemokines and pro-inflammatory cytokines [210–214].

**Fig. 4:**
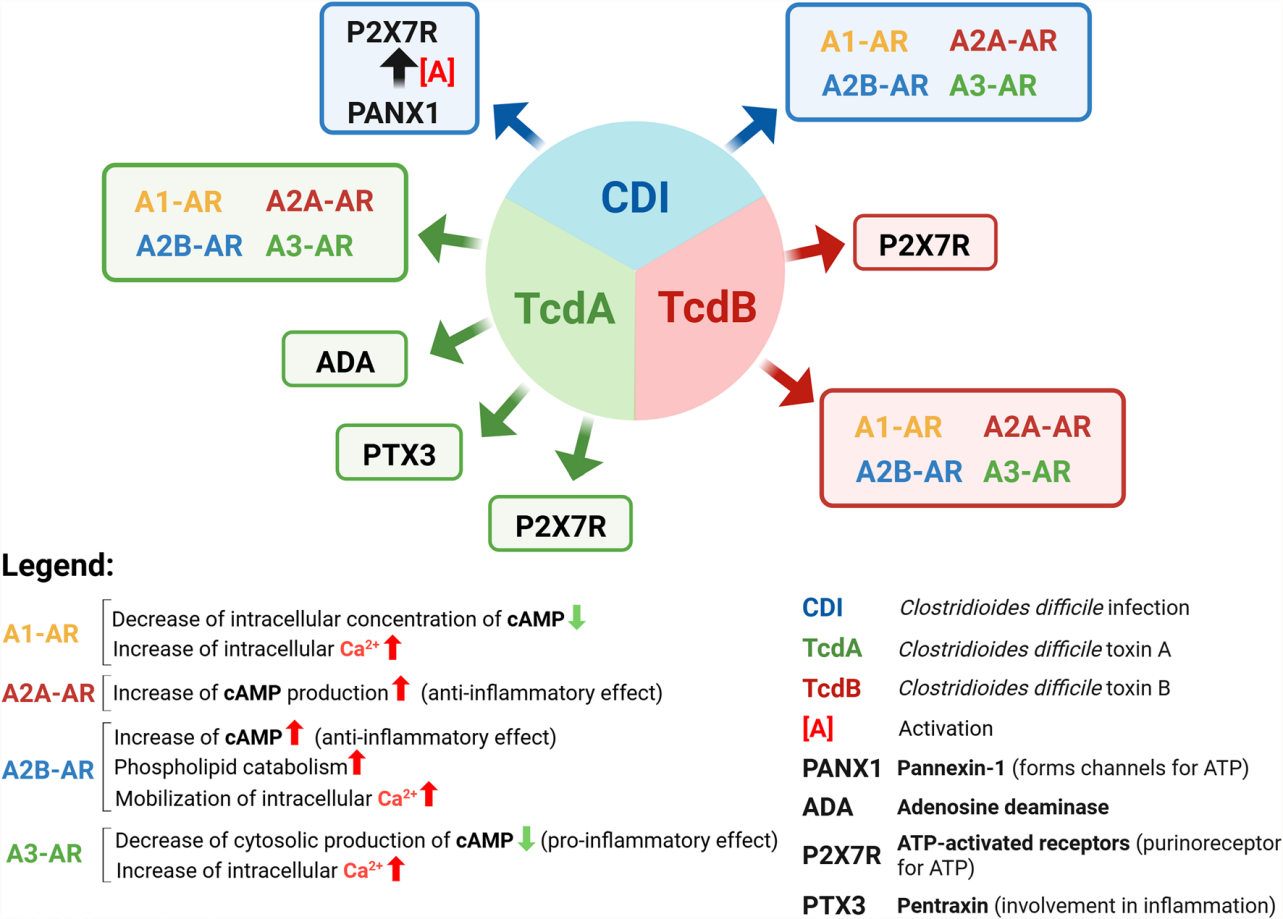
Interactions between *C. difficile* infection and its TcdA and TcdB with Adenosine system. Both TcdA and TcdB are able to interact with the four ARs as demonstrated in vitro and vivo. This also occurs during CDI in vivo, probably due to the action of the Tcds produced but not exclusively. The main effects of these interactions are reported in the legend (left side). Furthermore, TcdA interacts with P2X7R and PTX3 involved in inflammation and with ADA involved in the regulation of ADO levels, while TcdB is less involved in the inflammatory response and interacts only with P2X7R. In vivo CDI activates Panx1 which, by forming channels for ATP, is able to activate P2X7R. "Created in BioRender https://BioRender.com/ak31a9n"

Of interest, Tcds are capable of interacting with the ARs, influencing the course of infection (Fig. 4) [215–218].

### Molecular structure of Tcds

TcdA and TcdB are multifunctional proteins for: a) the complex molecular organization of their domains, b) the conformational changes due to variations in pH and after interaction with ligands [210–214], and c) the contribution of intrinsically disordered regions to their functionality [219]. TcdA is formed by 2710 amino acids (~ 308 kDa), while TcdB is by 2366 amino acids (~ 270 kDa). They have 51% sequence identity and 66% sequence similarity [210–214]. Tcds have an analogous glucosyltransferase enzymatic activity and a multi-domain structure, composed of 4 domains, referred to as the ABCD model (Domain A: Biological Activity; Domain B: Binding; Domains C: Cutting; Domain D: delivery) [210–214]. Domain A: matches with the N-terminal glucosyltransferase domain (GTD) that modifies the cytoskeleton by monoglucosylation of small GTPase of the Rho family. Domain B: matches with the C-terminal-located receptor-binding domain (RBD) that includes “the combined repetitive oligopeptide (CROP) domain”, which represents a relevant part of the “receptor-binding domain”. Domain C: matches with the “autoprocessing domain (APD) or cysteine protease domain (CPD), which mediates the autocatalytic cleavage. Domain D: matches with the “translocation/pore-forming domain or delivery domain” that mediates the translocation of the catalytic domain into the cytoplasm from the endocytic vacuole. This domain is also involved in receptor binding on target cells by Tcds [210–214].

### Receptors for Tcds and binding mode

TcdA binds two protein receptors expressed in the cell plasma membrane [211, 212, 220] The first TcdA receptor is the sucrase-isomaltase, a glycoprotein expressed in the brush border of small intestine [211, 212, 221] but not expressed in the human colonic epithelium and in various cells and tissues that are anyways susceptible to TcdA, indicating the presence of other types of receptors recognized by TcdA [211, 212, 221]. The second is glycoprotein 96 (gp96), a heat shock protein family member [211, 212, 222]. However, TcdA binds other receptors, because cells that did not express the gp96 receptor are only partly resistant to TcdA activity [211, 212, 221]

TcdA also binds to cell surface-associated oligosaccharides behaving like a lectin or members of the low-density lipoprotein receptor (LDLR) family [223–226]. Further, the TcdA also binds structures containing beta-galactosidase, which implies the binding capacity to Lewis A and GALB1-4 GLC NAC core structures [223–225]. The full glycan binding profile of TcdA has been localized to the RBD, where CROPs are believed to be responsible for interaction with carbohydrates expressed in the cell membrane [223–225].

The receptors for TcdB are three proteins expressed in the cell plasma membrane [211, 212, 220]. The first is chondroitin sulfate proteoglycan 4 (CSPG4) [220, 226, 227] the region involved in the binding is localized at the N-terminal of CROPs [220, 227, 228]. However, the receptor binding is not restricted to this Tcd domain, because it seems that the CROPs participate in but are not necessary for the binding to host cells [220, 228, 229]. CSPG4 is mainly expressed in the intestinal subepithelial myofibroblasts, but not on the epithelium surface [220, 226, 228, 230]. The second TcdB receptor is poliovirus receptor-like protein (PVRL3) defined also NECTIN3. The third TcdB receptors are the frizzled proteins 1, 2, and 7 (FZD1,2,7) [220, 228, 229, 231]. PVRL3 and FZDs are colonic epithelial receptors for TcdB because they are expressed on the surface epithelium of the human colon [220, 228, 229]. PVRL3 or FZDs are bound by TcdB using regions that extend beyond the CROPs [220, 228, 229]. It is still unknown if PVRL3 and FZDs bind TcdB in diverse regions or contend for the same region, while the binding to CSPG4 and to FZD occurs independently and in an additive manner [220, 231]. Thus, TcdB can bind membrane receptors with different molecular regions of its structure.

Furthermore, similar to TcdA, TcdB binds cell surface-associated oligosaccharides, behaving LDLR family [223, 226]: therefore, TcdB has a broader receptor specificity than TcdA, because it is able to bind sialylated and mannobiose glycans [223, 226]. This allows TcdB to have a wider range of target cells in the gastrointestinal tract compared to TcdA, and also a broader tissue tropism because TcdB has multiple lectin sites.

### Effects of Tcds

Tcds in vitro inactivate Rho-GTPase by glucosylation inducing so cytopathic effects (early cytoskeleton disruption, cell rounding, cell cycle arrest) and cytotoxic effects by apoptosis, necrosis and to a minor extent by pyknosis or pyroptosis [210–212, 232–236]. Furthermore, Tcds cause the production/secretion of chemokines and proinflammatory cytokines [210–213]. Fettucciari et al. demonstrated that enteric glial cells (EGCs) surviving TcdB-induced apoptosis [232] become senescent as a survival response to TcdB stressor stimulus [237]. Tcds in vitro induce also glucosyltransferase-independent effects, and Tcds at high concentrations mainly induce cell death (cytotoxic effect) by necrosis, pyknosis, and apoptosis that are independent by Tcd-autoprocessing or -glucosyltransferase activities [210, 211, 234–236, 238]. The effects of Tcds in vivo that are glucosylation-dependent are at the basis of the mechanism of pathogenesis and are cytoskeletal disruption, focal adhesion disassembly, and tight junction disruption, leading to severe edema, intense destruction of the mucosa, haemorrhage, and accentuated tissue inflammation with neutrophil infiltration and production of COX-2, prostaglandin E2, and inflammatory cytokines such as TNF-α and IL-1β, IL-6, and IL-8 and recruitment of other immune cells, which overall elicit watery diarrhoea [196, 210–212, 236, 239]. Tcds, by causing damage to the epithelium and to the deeper layers of the mucosa stimulate the host inflammatory and immune responses by which the immune system attempts to constrain the dissemination of intestinal bacteria into the circulation. However, an excessively strong and prolonged inflammatory response during CDI can be damaging to the host and contribute to the severity of CDI, by increasing the severity of tissue damage and the probability of lethal disease outcomes [210–212]. Moreover, CDI causes intestinal inflammation, and increases the production of ADO, thus initiating a complex interaction, the outcome of which decides the severity and progression of CDI or its containment.

## TCD interaction with the adenosine system

### Tcds and panx

Tcds have profound effects on the Panx1 and P2X7R systems. Indeed, they activate Panx1 in EGCs in vitro and upregulate P2X7R expression, parameters assessed at 18 h, before the profound alterations leading to cell death begin. The key element in this process is the release of eATP by the open channels of Panx1 which activates P2X7R. Thus, the activation of P2X7R occurs as a consequence of the direct activation of Panx1 by Tcds. This causes an intracellular Ca^2+^ increase that activates calpain and other calcium-dependent proteins. Calpain in turn activates initiator and effector caspases, resulting in cell death by apoptosis [240–243]. In this cascade of activation events, there is also the translocation of NF-κB and the induction of IL-6 expression [160].

In vivo, CDI in mice confirms that in the colon *C. difficile* induces an increase in Panx1-3 days after infection and that its inhibition with a Panx1 inhibitor, both in vitro and in vivo, protects against apoptosis, while IL-6 expression remains unaffected. Thus Tcds, by opening Panx1 channels, induce cell death by releasing eATP which activates purinergic P2X7R receptors that also respond by inducing an increase in Ca^2+^ released by the ER. The increase in Ca^2+^ activates calpain and other Ca^2+^-dependent proteins. Calpain in turn also activates initiator and effector caspases resulting in apoptosis. The stimulation/activation circuit of P2X7R by eATP can be self-sustaining because dead cells release eATP which then continues to increase P2X7R activation resulting in increased cell death. Moreover, as P2X7R is expressed in epithelial cells, neurons, macrophages, and T lymphocytes, this phenomenon of inflammation and activation of apoptosis by Tcds through P2X7R can become relevant during CDI, even in areas of the colon where the concentration of Tcds is not sufficient to induce cell death directly through its cytotoxic action, i.e. in areas at the edge of CDI action [160].

In a mouse model of ileitis, induced by TcdA, after 4 h exposure of the ileal loop to TcdA, there is an increase in P2X7R gene expression in myenteric neurons. In addition to neurons, EGCs, T lymphocytes, MCs, and DCs also have an expression of P2X7R bases. This increase in P2X7R in the context of Panx1 channel opening and eATP release induces myenteric neuron death and also increases S100B synthesis following the release of proinflammatory mediators, contributing to tissue damage, inflammation, and cell death in the ileum. S100B acts mainly as a pro-inflammatory mediator which, when it reaches high concentrations as in the case of CDI, activates NF-κB. The eATP released by dead cells increases the activation of P2X7R, which also promotes the secretion of the proinflammatory cytokines IL-1β, IL-6, TNF-α, and KC. Inhibition of P2X7R in this model overall reduces these events, i.e. cytokine release, cell death, S100B synthesis, and loss of myenteric neurons, confirming the centrality of this purinergic receptor. Indeed, P2X7R regulates the main pathways of cell death, apoptosis, necrosis, pyroptosis, and autophagy. Since cell death, in turn, induces the release of eATP from dead cells, the activation of P2X7R by TcdA could originate a self-feeding circuit because the released eATP increases the activation of P2X7R, which continues to promote the persistence of the proinflammatory cytokine cascade also by increasing the activation of NF-κB. The fact that in individuals with Crohn’s disease, the level of P2X7R increases suggests that its upregulation is a common phenomenon in inflammatory conditions. This could explain the increased severity of CDI in IBD, resulting in increased cell death [244].

### Tcds and ADA

In a mouse model of the ileal loop, TcdA induced a marked increase in ADA. Since ADO has anti-inflammatory effects, it is to be expected that the TcdA-induced increase in ADA activity would increase ADO degradation, resulting in increased leucocyte recruitment and potentiation of the inflammatory response and tissue damage. All this suggests a possible role for ADA in the pathogenesis of TcdA-induced inflammation. It is also possible that ADO, acting on the A2A-AR receptor present in the cells of the ileum, inhibits the production of pro-inflammatory cytokines and the production of chemokines such as TNF-α, IL-8, IL-6. Thus, the increase in ADA and subsequent decrease in ADO reduces the protective effect of ADO, on a key aspect of the inflammatory response. Indeed, the A2A-AR agonist, ATL313, reduces TcdA-induced mucosal ileal cell death [245]. Further confirmation of the role of increased ADA in cell death during inflammation occurs in a TcdA-induced model of gastroenteritis in mice. In fact, inhibition of ADA by EHNA after 3 h of infection with TcdA in the ileum of mice reduces tissue damage, neutrophil infiltration, and the level of the pro-inflammatory cytokines TNF-α, IL-1β, as well as the expression of NOS2, NF-κB and PTX3. In conclusion, TcdA increases ADA activity which reduces ADO availability. Since ADO, through stimulation of A2A-AR, reduces some of the relevant effects of TcdA which are due to TNF-α production, inflammation, and cell death, activation of ADA by TcdA reduces the protective role of ADO [246].

### Tcds and PTX3

In a mouse model of enteritis induced in C57BL6 or of the ileal loop, TcdA induces an increase in PTX3, which is reduced by an ADA inhibitor that naturally inhibits the inflammatory response. PTX3 is produced locally at the site of infection and inflammation by a variety of cell types including fibroblasts, endothelial cells, mononuclear phagocytes, and DCs in response to pro-inflammatory signals such as TNF-α, IL-1β, Toll-like receptor agonist [246].

## TCD interaction with the adenosine receptors and ecto-enzymes

Once ADO becomes extracellular or produced outside the cell, it binds to its four ARs in surrounding cells with different consequences depending on the type of AR involved as described in detail above [3, 13, 35, 68, 92, 93, 138, 146, 191]. In fact, the main effects in synthesis are: A1-AR increases the pro-inflammatory response in PMNs, A2A-AR decreases the inflammatory response in PMNs, A2B-AR modulates inflammatory cytokines and adhesion molecules, and induces MC activation, A3-AR regulates immune functions, MC activation and regulation.

Since, Tcds profoundly alter the homeostasis of the ADO system, as described in detail below (Fig. 4) [123, 215–218, 245–247], acting at the level of its generation, modulating the expression and regulation of ARs and finally interacting with the ARs, can affect several biological effects which all together could influence the course of CDI.

### Tcds and A1-AR

TcdA in vitro induces A1-AR expression in IE after 24 h (late expression), while in an ileal loop model, 3 days after infection increases the expression of A1-AR [216, 246].

This AR activated by ADO in PMNs increases the pro-inflammatory response. Its induced expression in vitro or in vivo in non-immune cells could contribute to the increase of the pro-inflammatory response that has a bivalent role in CDI, because on one hand it increases the anti-bacterial response, on the other hand the inflammatory response damages the tissue favoring the persistence of CDI.

### Tcds and A2A-AR

In EGCs, both TcdA and TcdB upregulate the expression of A2A-AR at 12 h that persisted at 18 h [215]. A similar result occurs when infecting HCT-8 cells, in which Tcds induce an early expression of this receptor at 6 h and a late expression at 24 h [216]. The ability of Tcds to influence the expression of this receptor was confirmed in various models in vivo. Indeed, in an ileal loop model in mice, TcdA three days after infection increased the expression of A2A-AR [246]. Moreover, CDI induced a progressive increase in this receptor for up to three days [216]. A2A-AR plays an important role in CDI because its deletion or inhibition in a mouse model of infection reduces survival as a consequence of increased toxicity and the inflammatory response characterised by increased TNF-α and IFN-γ [218]. However, in an ileal loop model, the activation of A2A-AR slightly reduced the effects of CDI, which may be because the initial effects of CDI are at the level of the intestinal epithelium where little A2A-AR is expressed [217].

TcdA increases the activity of ADA, which reduces the amount of available ADO, thus reducing its anti-inflammatory activity; at the same time, it increases the expression of A2A-AR and also A1-AR.

However, since ADO acts predominantly on the anti-inflammatory A2A-AR receptor and less on the A1-AR receptor, the reduction of ADO causes the anti-inflammatory response to decrease, and thus there is an increase in inflammation and its consequences [217].

All this favors the persistence and deepening of CDI, since it continues the damage of the epithelium and the underlying cell layers. This represents one of the key events in the pathogenesis of CDI because the persistence of inflammation manipulated through increased ADO activity creates an irreversible situation in which CDI is no longer kept under control.

### Tcds and A2B-AR

This receptor is the main ADO receptor expressed in the intestinal epithelium, mainly in the cecum and colon [217]. TcdA and TcdB upregulate the expression of A2B-AR in HCT-8 cells at 2 h until to 6 h [216, 217]. Furthermore, in EGCs, TcdA and TcdB induces downregulation of the expression of this receptor at 12 h that persisted at 18 h [215]. In an in vivo model, CDI causes a progressive increase in A2B-AR expression for up to three days that progressively ends at 7 days [216]. In an ileal loop model, blockade of A2B-AR is followed by a strong reduction of the inflammatory response and damage to the intestinal epithelium induced by TcdA [217].

In vitro gene knockout or in vivo deletion of A2B-AR reduces cell death, thus indicating a critical role of this receptor in Tcds-induced cytotoxicity in EGCs. Indeed, attenuation of this receptor reduces Tcds-induced caspase 3/7 activities [215]. Since blocking A2B-AR inhibits apoptosis, but simultaneously increases IL-6 expression, this cytokine may contribute to the anti-apoptotic effect that follows after A2B-AR inhibition.

### Tcds and A3-AR

TcdA and TcdB induce an increase in the A3-AR receptor in EGCs at 12 h that persisted at 18 h [215, 246]. Agonists of this receptor reduce apoptosis induced by the two Tcds while blocking this receptor does not affect apoptosis. When one summarises the effects of Tcds on EGCs taking into account that ADO acts on all expressed ARs, which are each regulated differently and have different functions, A2A-AR and A3-AR are upregulated, while A2B-AR is downregulated. Upregulation of A2A-AR and downregulation of A2B-AR in EGCs are protective events towards Tcds [215].

### Tcds and CD73

TcdA and TcdB increase after 8 h the expression of CD73 in an in vitro model of infection of the T84 colonocyte cell line. However, the cells at 24 h have already been profoundly altered by progressive cell death [247]. Also, in an ileal loop model in vivo, Tcds increase the expression of CD73. Activation of this 5'-nucleotidase, among other events, also leads to the production of ADO, which plays a rather complex role with regard to infection. On the one hand, it reduces inflammation and thus tissue damage, playing a protective role, but on the other hand, the reduction of the inflammatory response and thus of natural and adaptive immunity favours CDI. In fact, the prevalence of the protective effects of CD73 activity is demonstrated by the inhibition of CD73 itself, which causes an increase in the toxic effects of CDI. Finally, CDI-induced hypoxia, which stabilises the HIF1 protein α also contributes significantly to the increase in CD73. However, the most relevant event, as demonstrated in vitro, is the ability of Tcds to directly induce CD73 expression [247]. This implies that CD73 increases ADO production and thus reduces the proinflammatory response.

## Conclusions

*C. difficile* through its two main Tcds, TcdA and TcdB is able to interact with the ADO system and manipulate it profoundly (Fig. 4) [215–218, 245]. In fact, both Tcds are able to induce the expression of the four ARs for ADO on various types of colon cells and could induce/modulate the ARs expression on various types of innate and adaptive immunity cells.

Furthermore, by acting on the enzymes that determine ADO formation, chemical transformation and inactivation, Tcds are able to influence ADO extracellular levels [246, 247].

However, the picture of the interaction between CDI and the ADO system is extremely complex because the reduction of ADO favors the pro-inflammatory response that contributes to damage to the colon epithelium with consequent deepening of the infection and expulsion through the feces of *C. difficile* into the surrounding environment. At the same time, the inflammatory response, characterized in the first phase by the recruitment of cells of innate immunity, contributes to the containment of the infection.

Added to all this is the fact that each type of immune cell expresses ARs whose extent of expression is modulated by the interaction with Tcds, thus making their response no longer linear according to homeostatic rules, but profoundly and variably influenced by the quantity and quality of the expressed ARs, modulated by Tcds.

Therefore, *C. difficile* with its Tcds in the most acute zone of ​​inflammation that allows the infection to deepen, benefits from a reduction in ADO levels, while in the peripheral zones of infection a higher ADO level reduces the inflammatory response that favors the progression of the infection initially only at the level of the epithelia.

At present, the potential therapeutic target of ADO remains difficult to define, because of its hypothetical administration, if on the one hand reduces the negative consequences of the inflammatory response, on the other hand it also reduces the antimicrobial efficacy of this response.

However, ADO could be used during a specific antibiotic therapy for *C. difficile*, whereby the inflammatory effects would be greatly attenuated in a context where the bacterial load is progressively brought under control by the therapy.

Since it is often difficult to eradicate *C. difficile* even after one or more courses of antibiotic therapy, the use of ADO in conjunction with antibiotic therapy could greatly reduce the side effects of antibiotic therapy.

This could also reduce the subsequent incidence of Irritable Bowel Syndrome (IBS) and IBD associated with CDI.

## Data Availability

Not applicable.

## Abbreviations

5-AMP: 5-Adenosine monophosphate
5-IMP: 5-Inosine monophosphate
AC: Adenylyl cyclase/Adenylate cyclase
ADA: Adenosine deaminase
ADO: Adenosine
ADP: Adenosine diphosphate
AK: Adenosine kinase
AMP: Adenosine monophosphate
AP: Alkaline phosphatase
ARNO: ADP-rybosylation factor nucleotide site opener
ARs: Adenosine receptors
ATP: Adenosine triphosphate
Breg: Regulatory B lymphocytes
*C. difficile*: *Clostridioides difficile*
cAMP: Cyclic adenosine monophosphate
CD203α: Ectonucleotide pyrophosphatase/phosphodiesterase family member 1
CD38: Cyclic ADP ribose hydrolase
CD73: Endo-5'-nucleotidase
CDI: *Clostridioides difficile *infection
CDT: *Clostridioides difficile* transferase
cN-I: Cytoplasmatic-5-nucleotidase-I
CNT: SCL28 family of cation-linked concentrative nucleotide transporters
COX-2: Cyclooxygenase-2
CREB: CAMP-responsive element-binding protein
CSPG4: Chondroitin sulfate proteoglycan 4
DAMP: Damage-associated molecular pattern
DARPP-32: Dopamine- and cAMP-regulated neuronal phosphoprotein
DC: Dendritic cells
eATP: Extracellular adenosine triphosphate
ECTO-ADA: Ecto-adenosine deaminase
EGCs: Enteric glial cells
ENT: SLC29 family of energy-independent equilibrative nucleoside transporters
ER: Endoplasmic reticulum
FAE: Follicle-associated epithelium
GABA: γ-Amino-butyric-acid
GAP: Goblet cell associated antigen passages
Gi1: G protein of the inhibitory type
GPCRs: G-protein-coupled receptors
G-protein: GTP-dependent protein
Gs: G-stimulatory protein
GSK-3β: Glycogen synthase kinase-3-beta
IDO-1: Indoleamine 2,3-dioxygenase 1
IE: Intestinal epithelia
IECs: Intestinal epithelial cells
Foxp3: Forkhead box P3
IFN-γ: Interferon γ
IL-2: Inteleukin-2
IL-4: Inteleukin-4
IL-6: Inteleukin-6
IL-8: Inteleukin-8
IL-10: Inteleukin-10
IL-12: Inteleukin-12
IL-13: Inteleukin-13
IMPDH: Inosine-5-monophosphate dehydrogenase
iNOS: Inducible nitric oxide synthase
IP3: Inositol 1,4,5-trisphosphate
ISCs: Intestinal stem cells
LAG-3: Lymphocyte activation gene 3
MAPK: Mitogen-activated protein kinase
MCs: Mast cells
MIP-1: Macrophage inflammatory protein-1
MMP-1: Matrix metallo proteinases 1
MMP-3: Matrix metallo proteinases 3
MMP-9: Matrix metallo proteinases 9
MMP-12: Matrix metallo proteinases 12
NAD +: Nicotinamide adenine dinucleotide
NADPH: Nicotinamide adenine dinucleotide phosphate hydrogen
NF-κB: Nuclear factor kappa-light-chain-enhancer of activated B cells
NO: Nitric oxide
NPP: Nucleotide pyrophosphatase/phosphodiesterase
NTPDase1, CD39: Ectonucleoside-triphosphate-diphosphohydrolase-1
Panx1: Pannexin 1
PD1: Programmed cell death protein 1
PK: Protein kinase
PKA: CAMP-dependent kinase
PLC: Phospholipase C
PMN: Polymorphonuclear neutrophil granulocytes
PRR: Pattern recognition receptors
ROS: Reactive oxygen species
SAH: S-adenosyl homocysteine
TcdA: *Clostridioides difficile* Toxin A
TcdB: *Clostridioides difficile* Toxin B
TGF-β: Trasforming Growth Factor Beta
TIGIT: T cell Ig and iTIM domain
TIM-3: T cell Ig mucin domain-3
TJs: Tight junctions
TNF-α: Tumour necrosis factor alpha
TRAX: Translin-associated protein X
USP4: Ubiquitin-specific-protease-4
UTP: Uridine-5′-triphosphate
VEGF: Vascular Endothelial Growth Factor
